# Immunomodulatory Effects of Gold Nanoparticles: Impacts on Immune Cells and Mechanisms of Action

**DOI:** 10.3390/nano15151201

**Published:** 2025-08-06

**Authors:** Khadijeh Koushki, Prapannajeet Biswal, Geraldine Vidhya Vijay, Mahvash Sadeghi, Sajad Dehnavi, Ngoc Tuyet Tra, Sai Kumar Samala, Mahdieh Yousefi Taba, Arjun Balaji Vasan, Emily Han, Yuri Mackeyev, Sunil Krishnan

**Affiliations:** 1 Vivian L. Smith Department of Neurosurgery, University of Texas Health Science Center, Houston, TX 77030, USA; khadijeh.koushki@uth.tmc.edu (K.K.); prapannajeet.biswal@uth.tmc.edu (P.B.); geraldine.v.raja@uth.tmc.edu (G.V.V.); ngoc.tuyet.tra@uth.tmc.edu (N.T.T.); sai.kumar.samala@uth.tmc.edu (S.K.S.); arjun.b.vasan@uth.tmc.edu (A.B.V.); 2 Faculty of Medicine, Mashhad University of Medical Sciences, Mashhad 13944-91388, Iran; mahvashsadeghi8@gmail.com (M.S.); yousefi_t.m@yahoo.com (M.Y.T.); 3 Allergy Research Center, Mashhad University of Medical Sciences, Mashhad 13944-91388, Iran; dehnavis@mums.ac.ir; 4 Department of Biosciences, Rice University, Houston, TX 77251-1892, USA; egh5@rice.edu

**Keywords:** gold nanoparticle, inflammation, anti-inflammatory, innate immunity, adaptive immunity

## Abstract

Traditional anti-inflammatory medications—such as corticosteroids, biological agents, and non-steroidal anti-inflammatory drugs—are commonly employed to mitigate inflammation, despite their potential for debilitating side effects. There is a growing need for alternative next-generation therapies for symptomatic, unchecked, and/or detrimental inflammation with more favorable adverse effect profiles. The long history of use of gold salts as anti-inflammatory agents and the more recent exploration of gold nanoparticle (AuNP) formulations for clinical indications suggest that the targeted delivery of nanoparticles to inflammatory sites may be a promising approach worth investigating. Coupled with peptides that specifically target immune cells, AuNPs could potently counteract inflammation. Here, we provide an overview of the selective infiltration of AuNPs into immune cells and summarize their interactions with and impact on these cells. Additionally, we provide a comprehensive mechanistic summary of how AuNPs exert their anti-inflammatory effects.

## 1. Introduction

Inflammation is a protective biological immune system response. It is a rapidly cascading mechanism of innate immunity activated in response to chemical or physical stimuli, as well as biological agents. Its purpose is to combat invasion, facilitate tissue repair, and maintain homeostasis [1]. This process is intricate, tightly regulated, and involves various physiological, cellular, and molecular changes that are crucial for overall health and homeostasis. However, uncontrolled inflammation results in tissue injury and worsens various immunological and metabolic disorders. Additionally, inflammation-induced fibrosis, while important for maintaining structural integrity, can further impair tissue function [2].

The treatment of inflammatory disorders typically involves immunosuppressant or anti-inflammatory drug regimens for symptomatic treatment [3]. The fundamental role of underlying tissue inflammation in the development of these disorders underscores the importance of targeting inflammatory processes to aid diagnoses and therapy. More recently, there has been a focus on strategies that target the cellular mediators of inflammation, the innate immune cells, such as neutrophils, monocytes/macrophages, mast cells, dendritic cells (DCs), innate lymphoid cells (ILCs), and natural killer (NK) cells, aimed at reducing their migration to damaged and inflamed sites.

Nanoparticles (NPs) have emerged as next-generation drug delivery systems that allow for a high drug-loading capacity and the delivery of various therapeutic agents [4]. NPs can be designed to carry immunomodulators and antigens, allowing for precise homing to the immune system and the potential to target both adaptive and innate immune cells, thereby suppressing inflammation through multiple pathways [5]. Additionally, NPs can improve the pharmacokinetics and bioavailability of therapeutics, making them promising tools for enhancing disease treatments. Numerous nano-based drug studies have been published to date, with many currently undergoing clinical trials [6].

Among the multitude of NPs studied, gold nanoparticles (AuNPs) continue to garner increasing interest in their potential applications in immunotherapies. Their advantageous physicochemical properties, including tunable size, shape, and surface functionalization, relative thermal and chemical inertness and stability, and the long history of use of gold salts for medicinal applications make them attractive as potential anti-inflammatory agents [7,8,9]. Ongoing basic and animal studies investigating the immune-modulatory properties of AuNPs seek to document their efficacy in treating different forms of inflammation. Equally, evolving studies assess associated risks, undesirable effects, and variations in response profiles among individuals. Collectively, these studies benchmark the suitability of AuNPs for treating unchecked inflammatory conditions. In this review, we first examine the properties of AuNPs as drug carriers. Subsequently, we delve into the various mechanisms by which AuNPs regulate immune responses and inflammation and outline their interactions with individual immune cells, with a focus on their anti-inflammatory properties.

## 2. The Inflammatory Process

The primary characteristic of inflammation is the increased presence of leukocytes in the bloodstream and their migration into damaged or inflamed tissues. This process involves molecular mediators, immune cells, and blood vessels working together to eliminate the initial cause of cell injury, facilitate tissue repair and healing, and establish immune memory [10,11,12]. Monocytes play a pivotal role in responding to inflammatory injuries. They typically originate from progenitor cells in the marrow as classical or inflammatory monocytes and are then subsequently recruited to the sites of ensuing inflammation. There, they mature into inflammatory macrophages and dendritic cells (DCs) [13]. These cells, along with other resident cells—such as lymphocytes, mast cells, fibroblasts, and endothelial cells—in the injured or infected tissue, produce soluble mediators (e.g., complement, cytokines, free radicals, chemokines, vasoactive amines, and eicosanoids like prostaglandins). This results in the activation of local blood vessels, the promotion of inflammation, and the mediation of various associated functions. Granulocytes and neutrophils are similarly recruited from the bloodstream to inflamed tissues as part of the initial defense line and work in tandem with DCs, monocytes, and macrophages to complement and synergize with the myeloid lineage response [14,15]. While this phenomenon occurs naturally as a response to a stimulus, the excessive infiltration of cells becomes pathological and is evident in various diseases, such as viral or bacterial infections, strokes, traumatic injuries to the brain or spinal cord, and autoimmune diseases [16,17,18,19,20]. Depending upon the nature of the injurious stimulus, its persistence, and the resultant injury, inflammation can manifest in either acute or chronic forms.

### 2.1. Acute Inflammatory Response

Acute inflammation results from trauma, microbial invasion, or exposure to noxious compounds, radiation, and other injurious stimuli [1,21]. It involves the release of a multitude of soluble mediators, including acute-phase proteins (APPs), chemokines, and cytokines. These mediators recruit and activate leukocytes at the inflamed sites, particularly a significant number of neutrophils, which play a central role in the acute inflammatory response. Infiltrated neutrophils eliminate pathogens, subsequently amplifying the immune response and initiating inflammatory cascades to remove the inflammatory stimulus or damaged cells, thereby initiating the healing process [17,22]. Depending on the degree of injury, these cellular and molecular events of the acute-phase response are typically sufficient to resolve and minimize infection or injury and initiate healing processes.

### 2.2. Chronic Inflammation

Chronic inflammation is implicated in the pathogeneses of several diseases, including autoimmune disorders, metabolic syndromes, cardiovascular diseases, infectious diseases, neurodegeneration, and even cancer [23,24,25]. Chronic inflammatory diseases affect nearly 1 in 20 individuals and can manifest either as a systemic (e.g., systemic lupus erythematosus [SLE]) or tissue-localized (e.g., inflammatory bowel disease [IBD], psoriasis, and rheumatoid arthritis [RA]) disorder [26]. Unresolved acute inflammation, persistent inflammation, and the inability to eliminate tissue injury, often due to continued exposure to risk factors or an inapt immune response against self-tissue antigens, can propagate chronic inflammation. In chronic inflammation, active immune cell populations shift to a mononuclear phenotype, which drives tissue damage and fibrosis [1]. Prolonged exposure to stimulation leads to the generation and activation of other pro-inflammatory mediators that attack healthy cells and tissues, resulting in tissue degeneration and dysfunction, associated with pathological conditions [27].

### 2.3. Role of the Immune System in Inflammatory Responses

The host immune system detects danger signals through conserved motifs known as pattern recognition receptors (PRRs), expressed in both immune and non-immune cells, such as endothelial cells and tissue-resident sentinel macrophages. Upon activation, PRRs concurrently activate multiple critical intracellular signaling pathways, including the Janus kinase (JAK)-signal transducer and activator of transcription (STAT) pathway, mitogen-activated protein kinase (MAPK), and the nuclear factor kappa-B (NF-κB) pathway [10,28]. These responses often set off the inflammatory cascade by inducing the surface expression of adhesion molecules, including cadherins, selectins, and integrins, which are readily targeted with NPs due to their expression on vascular endothelial surfaces [29]. Additionally, they stimulate the release of various soluble mediators (chemokines and cytokines) such as monocyte chemotactic protein-1 (MCP-1) and granulocyte macrophage-colony stimulating factor (GM-CSF). This further leads to the generation and recruitment of innate inflammatory cells, such as monocytes and neutrophils, which mediate local responses, initially from the bone marrow into the peripheral circulation and subsequently to damaged or infected tissue sites. [30,31]. The secretion of these cytokines plays a pivotal role in the subsequent phases of the immune response and can lead to the production and release of pro-inflammatory mediators, including cytokines like interleukins (IL-1, IL-6, and IL-10), type 1 interferons (IFNs), and tumor necrosis factor-alpha (TNF-α) [32].

Neutrophils are the primary immune cells attracted to damaged sites, subsequently followed by monocytes, lymphocytes (including NK, T, and B cells), and mast cells [33,34]. However, the types of leukocytes infiltrating the infection site depend on the nature of the stimulus and their presence in different lesions. Infiltrated monocytes in damaged tissues can transform into DCs and tissue macrophages, which phagocytose pathogens, dead neutrophils, and cell debris, aiding in the tissue repair process. Furthermore, stimulated toll-like receptors (TLRs), a type of PRR, induce the functional maturation of DCs into potent antigen-presenting cells (APCs), efficiently initiating and controlling adaptive immune system responses. This recruitment and activation of B cells and cytotoxic T cells, in combination with IFN-γ, can induce M1 macrophage polarization, which is crucial for phagocytosis and the elimination of invading pathogens.

Monocytes and neutrophils promptly transmigrate into acutely inflamed tissues, where they transform into effector cells. While monocytes polarize to a pro-inflammatory M1 phenotype, activated neutrophils secrete neutrophil extracellular traps (NETs) and proteases, which further complement the functions of M1 macrophages. As inflammation resolves, NETs and proteases are gradually degraded, neutrophils undergo apoptosis, and macrophages engage in the efferocytosis of neutrophils and dead tissue. Further, macrophages transition from the M1 to M2 phenotype during this phase. Upon failure of resolution, persistently activated macrophages and neutrophils drive the pathogenesis of various vascular diseases. In the ensuing chronic inflammation, these cellular mediators activate and regulate platelets, as well as adaptive immunity, to establish an altered, pro-inflammatory homeostatic phase that inevitably results in tissue damage and remodeling [35].

**Figure 1:**
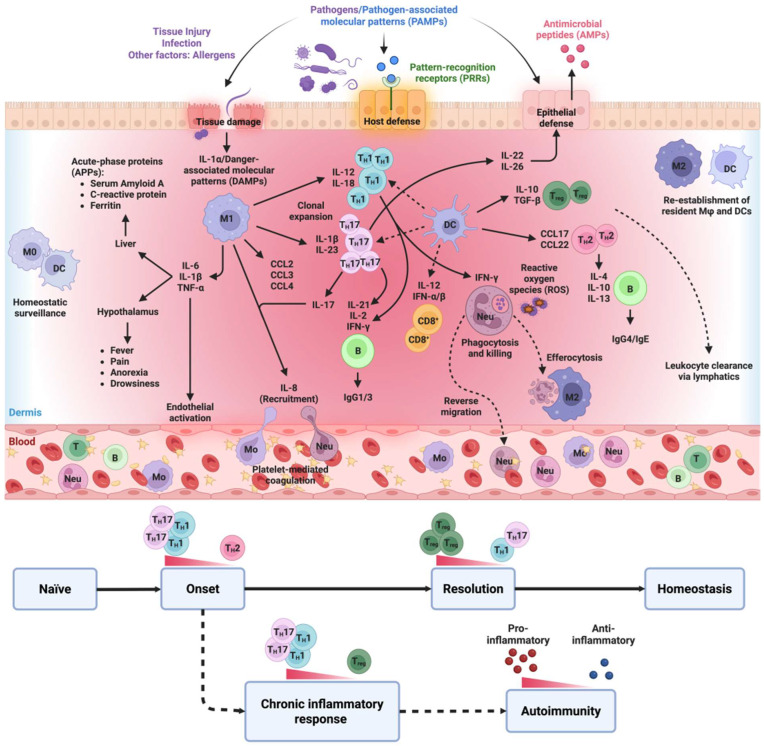
Schematic of cellular dynamics across the stages of inflammation. Depicts epithelial and immune sensing of pathogen-associated molecular patterns (PAMPs) and damage-associated molecular patterns (DAMPs) via pattern recognition receptors (PRRs). DAMPs released from injured host cells enter damaged tissues and are sensed by resident macrophages, triggering IL-1α–mediated activation. This initiates maturation of dendritic cells, cytokine-driven recruitment of neutrophils and monocyte-derived effectors, and antimicrobial peptides (AMPs) release. Macrophages and dendritic cells also activate naïve T and B cells in draining lymph nodes, leading to clonal expansion and differentiation of effector lymphocytes. The inflammatory response is amplified, followed by successful resolution involving clearance of pathogens, debris, and apoptotic cells, reverse migration of infiltrating cells, leukocyte egress via lymphatics, and reassembly of the tissue-resident immune niche. Failure to resolve may lead to sustained inflammation and development of chronic inflammatory response or autoimmunity.

Neutrophils, which primarily neutralize infective microorganisms in the body, can also harm host cells and tissues. As the key mediators of the inflammatory cascade, they program the release of localized factors to attract monocytes and DCs, while simultaneously programming APCs to activate T cells [17]. Macrophages, key effectors of the mononuclear phagocyte system, are critical in initiating, maintaining, and resolving the inflammatory process. They present antigens on their surfaces to act as APCs, undertake phagocytosis, and modulate the immune response by producing various cytokines and growth factors [36]. Mast cells, granulocytes residing on epithelial surfaces and in connective tissue matrices, also hold the potential to act as effector cells that trigger inflammatory responses. Upon activation, they release various inflammatory mediators, including cytokines, chemokines, histamine, proteases, prostaglandins, leukotrienes, and serglycin proteoglycans [37]. Studies have shown that platelets also influence both acute and chronic inflammatory processes, from infections to atherosclerosis, and their interactions with inflammatory cells possibly contribute to pro-inflammatory outcomes. Platelets may also induce the acute-phase response (APR) in some cases [38], which is then sustained and amplified by immune cells recruited to the sites of inflammation by releasing locally acting inflammatory mediators (Figure 1).

Inflammatory cells have the potential to inflict direct damage on tissues, but they can also intensify the inflammatory response by generating and releasing certain cytokines. Moreover, they influence the proliferation and activation of adaptive immune cells, thereby sustaining the persistence of the inflammatory reaction. Various stimuli trigger the activation of inflammatory cells, leading to the release of pro-inflammatory cytokines (IL-1β, IL-6, and TNF-α) along with pro-inflammatory proteins and enzymes. These molecules hold promise as potential biomarkers of disease diagnoses and prognoses and can influence therapy [39]. The inflammatory response is a meticulously orchestrated network of signaling pathways that govern numerous inflammatory mediators and cell types. These events are regulated by an array of locally and distantly acting extracellular mediators and regulators, which include cytokines, growth factors, eicosanoids (prostaglandins and leukotrienes), the innate complement system, and peptides. The excessive and prolonged activation of immune cells, including T and B lymphocytes, and the overexpression of master pro-inflammatory cytokines such as TNF-α, in conjunction with other mediators like IL-6, IL-1β, and IFN-γ, assume a central role in the pathogenesis of autoimmune inflammatory diseases [20,40].

## 3. Current Clinical Approaches to Inflammatory Diseases

There are various treatment options for inflammatory conditions, including broad immunosuppressive treatments like corticosteroids (e.g., glucocorticoids and mineralocorticoids) and non-steroidal anti-inflammatory drugs (NSAIDs), which have traditionally been used to reduce inflammation by inhibiting leukocyte function. However, they are associated with serious unwanted side effects [41]. More recently, there has been a shift towards generalized suppression through the delivery of antibodies, proteins, peptides, nucleic acids, and fluid infusion [42,43,44]. Host modulation therapy with anti-inflammatory agents is not a novel concept, and many drugs have been assessed for their potential in managing inflammatory conditions. The two key objectives in anti-inflammatory treatments are (I) suppressing overactive inflammatory responses to prevent self-harm and (II) restoring cell functions to a state of homeostasis.

In recent years, a category of drugs known as biological therapies (or ‘biologics’) has gained prominence, particularly for the treatment of autoimmune and other chronic inflammatory conditions. Biologics encompass several anti-cytokine agents that target pro-inflammatory cytokines or immune-competent molecules. These drugs selectively inhibit the activity of specific cytokines. Biologics are typically monoclonal antibodies that bind to the target cytokine. Examples include anti-TNFα blockers (such as adalimumab, certolizumab-pegol, etanercept, golimumab, and infliximab), which have significantly improved the management of autoimmune diseases. Anakinra, a recombinant IL-1 receptor antagonist; natalizumab, a specific monoclonal antibody used to prevent mononuclear leukocyte migration across the blood–brain barrier (BBB) into the brain parenchyma; rituximab, an anti-CD20 monoclonal antibody that depletes B cells; and tocilizumab, a humanized anti-IL-6 receptor monoclonal antibody, are also part of this group. Another group includes toll-like receptor (TLR) inhibitors, but this research is in its early stages, with safety concerns being a primary focus due to the fundamental importance of TLRs in infection and immunity [45]. However, despite their effectiveness as immune suppressants, these and similar treatments can have debilitating side effects, such as the reactivation of serious infections like tuberculosis, an increased risk of opportunistic infections, and the development of lymphomas and solid tumors [46]. Given these side effects associated with traditional and current treatments, the next generation of therapies should prioritize specific therapeutic approaches to enhance treatment efficacy, reduce inflammation, and improve symptoms while minimizing adverse effects on the body’s immune cells and tissues compared to current treatments.

Innovative strategies, including the use of NPs to target sites of inflammation, offer a promising alternative to mitigate some of these complications and enhance treatment outcomes [47]. Protein- and peptide-loaded NPs have been employed to target innate and adaptive immune cells (neutrophils, macrophages, DCs, and T and B cells) with the aim of suppressing inflammation [5]. Additionally, NPs are being developed to target immune cells and can carry substances like siRNA, among other pharmaceuticals, to influence cellular responses in a targeted fashion [48,49]. Despite encouraging results, experimental and regulatory complexities related to drug loading and stability, either on the particle surface or within, roadblock their translation to clinical settings [50]. The application of NPs for the treatment of inflammatory diseases can provide innovative solutions to help improve the efficacy of current immunosuppressive therapies while simultaneously mitigating the side effects associated with conventional therapies. Several studies support the enhanced effectiveness of NP-based drug delivery strategies compared to current therapies in treating inflammatory and autoimmune diseases. Nanomedicine can also reduce prescribed doses and, consequently, the side effects of traditional therapies.

### Nanotechnology: Definition and Diverse Applications

Nanotechnology brings about significant advantages for human health by harnessing various nanomaterials with unique optical, electronic, and magnetic properties. In the 21st century, nanotechnology and nanoscience have emerged as pioneering technologies that impact the forefront of medical diagnostics and therapeutics. This influence spans from applications like biosensors, drug delivery, microfluidics, and microarray tests to tissue engineering. Nanomaterials are essentially tiny materials, with sizes ranging from 1 to 100 nanometers (nm), that can be fashioned from various materials such as polymers, metal particles, and carbon particles, among others. Nano-sized particulates can be broadly categorized as organic or inorganic based on their composition. Owing to their extraordinary biological, chemical, electrical, physical, magnetic, and optical properties, these nanoscale materials find rapid applications across biomedical applications such as diagnostics, therapeutics, bioelectronics, biosensors, and biochips, among others [51,52,53].

In comparison to conventional therapies, nanomedicine offers notable clinical benefits, boasting a superior therapeutic efficacy and reduced off-target toxicity. Nanomedicine involves the application of these nanoscale-structured materials in medicine, combined with biotechnology, for purposes ranging from diagnosis and prevention to therapies implemented across a wide spectrum of diseases, thereby enhancing human health [54]. Thanks to their nanoscale size and well-designed structure, NPs can be administered into the circulatory system, where they encounter multiple biological barriers before reaching their target sites, such as blood vessels and cell membranes. Subsequently, they are redistributed throughout various organs and subcellular locations relevant to drug action [55], making them potentially valuable in immunotherapies. Furthermore, NPs possess a large surface area-to-volume ratio due to their small size, enabling them to efficiently bind, adsorb, and carry therapeutic agents like drugs, proteins, antigens, RNA, DNA, and imaging agents [56]. Consequently, nanomedicines have garnered significant attention for their ability to deliver therapeutic agents. Drugs can be attached to or encapsulated within NPs to enable precise delivery to desired cell types or tissues, and NPs can also be designed to allow for controlled drug release [57].

There are various types of NPs, which, based on their composition, employ diverse materials in their synthesis. These materials encompass metallic oxides (e.g., iron oxide and titanium oxide), metalloids (e.g., amorphous silica and crystalline silica), organic biodegradable materials (e.g., lipids, polysaccharides, and polymeric matrices), inorganic metals (e.g., gold, silver, and carbon), liposomes, and virus-like particles (VLPs) [58]. These materials often share certain characteristics such as size range, hydrophilic properties, and charge attributes, which make them suitable carriers for drug delivery. However, the effectiveness of these nanostructures as efficient drug delivery vehicles is variable and is influenced by factors like size, shape, and other inherent chemical and biophysical characteristics.

Utilizing NPs as carriers for therapeutic agents offers several unique biological advantages over conventional formulations. For instance, (I) NPs can penetrate various physiological barriers, including the blood–brain barrier (BBB), enhancing drug transport across biological membranes, (II) they can enhance the specificity of drug molecules through targeting and co-delivering drugs and targeting agents, thereby reducing the required drug dose, (III) NPs can improve the plasma half-life, pharmacokinetics, and bioavailability of drugs, shielding them from enzymatic degradation and sustaining drug release over time, (IV) they can enable the delivery of multiple diagnostic and therapeutic agents concurrently, known as “theranostic” agents, which helps to overcome multidrug resistance mechanisms, (V) NPs can offer controlled, sustained drug release, and (VI) they can enhance the delivery of poorly soluble compounds through more suitable administration routes, ultimately reducing toxicity and increasing efficacy [59].

NP-based therapies hold great promise for pharmaceutical industries by significantly improving treatment efficacy while minimizing potential drug toxicity compared to conventional therapies. To date, several nano-drugs have received approval for routine clinical use and are available on the market, particularly for tissue imaging, cancers, autoimmune diseases, and infectious diseases. Many others are currently undergoing clinical trials [6]. Despite these advantages, it is important to consider how NPs interact with the immune system, as they can have complex effects, raising questions about their safety. NPs can act as a “double-edged sword”, offering both positive and negative health effects upon exposure. Consequently, three key immunological concerns must be addressed when engineering nanomaterials for in vivo applications, including (I) immune-mediated destruction or rejection, (II) immunotoxicity, and (III) immuno-safety or immune-compatibility [60]. While extensive clinical data is needed to fully exemplify the advantages and disadvantages of these vehicles, current evidence suggests that NPs can influence the immune system and impact disease processes such as allergies, inflammation, and tumors in animal models [50].

Among the various inorganic NPs, iron oxide, silver, and gold NPs show promise for various applications. Some gold and silver NPs have been approved for clinical use, while others are still undergoing pre-clinical trials. Metallic NPs, like gold and silver, possess unique properties such as surface plasmon resonance (SPR), which sets them apart from organic NPs like liposomes and micelles. They offer numerous advantages in drug delivery, including ease of synthesis, high drug loading capacity, improved bioavailability, low inherent toxicity (AuNPs), controlled drug release, superior storage stability compared to liposomes and micelles, and compatibility with certain organic solvents, unlike polymer-based NPs [56]. NPs can interact with various components of the immune system, including cells, proteins, receptors, and more, affecting cell signaling pathways and potentially causing unpredictable immune responses and adverse outcomes [61,62]. Therefore, a clear and nuanced understanding of the influence and modulation of the immune system by NPs is critical for assessing potential risks when developing new nanomaterials. In this context, NPs can be designed to precisely control the activation, function, and tissue infiltration of immune cells such as macrophages to facilitate inflammation resolution. Specifically, NP properties such as charge, hydrophilicity/hydrophobicity, size, and the steric effects of NP coatings can be tailored to achieve compatibility with the immune system [63,64]. Among the various materials used, gold NPs have garnered attention for their potential application in immunotherapies due to their advantageous physicochemical properties, which can be further easily modified in terms of shape, size, and functionalization, including variants like nanorods, nanotriangles, nanocages, and nanostars [7,8,9,65].

## 4. Gold Nanoparticles (AuNPs): Characterization and Multifaceted Applications

**Figure 2:**
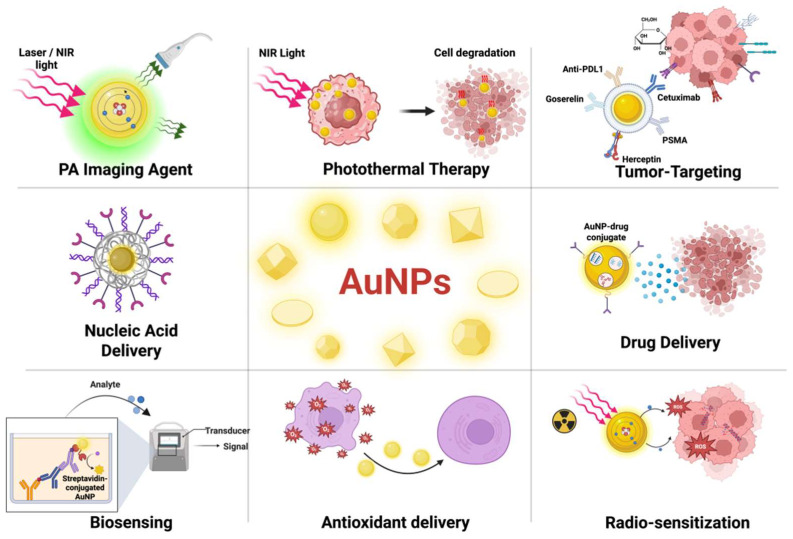
Diverse biomedical applications of gold nanoparticles (AuNPs). The unique properties of AuNPs allow for diverse biomedical applications, such as photothermal therapy, biosensing, molecular imaging, and radiosensitization. AuNPs can also be used for targeted delivery of various substances, such as nucleic acids, antioxidants, or drugs to a specific site. The efficacy of AuNPs in these applications depends on the particle shape, size, and environment. NIR = near infrared, PA = photoacoustic, PD1 = programmed death receptor-1, PSMA = prostate-specific membrane antigen, ROS = reactive oxygen species.

Today, AuNPs find extensive use in targeted drug delivery, encapsulation, cancer diagnostics, and therapies [66]. Among the various nanomaterials available, AuNPs have garnered significant attention in the field of nanomedicine due to their unique physical, chemical, optical, and immunological properties. These NPs offer several notable advantages, including ease of synthesis with an adjustable size (ranging from a [60] fullerene 1 nm size to over 100 nm), biocompatibility, minimal toxicity of unmodified particles in humans, distinct surface properties, SPR-mediated photothermal heating capability, multifunctional potential, and a high chemical stability, making them versatile across numerous applications [67,68,69]. The SPR bands on the surface of AuNPs enable them to convert light into heat, a property used to eliminate cancer cells by generating heat [70]. Furthermore, their negatively charged surface facilitates functionalization with various biomolecules like proteins, peptides [71], genetic materials (DNA, RNA, siRNA, etc.), drugs, and targeting ligands, making them ideal for drug delivery and facilitating novel combination therapies [72,73]. These properties position AuNPs as highly promising materials for diverse biomedical applications, such as diagnostic tracers, drug carriers, and for gene therapy, radiotherapy, photothermal therapy, biosensing, and molecular imaging (Figure 2), among others [29,74,75,76,77,78].

AuNPs have proven effective for the delivery and controlled release of chemical and biological agents for diverse therapies, including anti-cancer drugs (PEGylated Cisplatin tethered to gold nanospheres) [79], anti-oxidants (Glutathione-coated AuNPS for targeted delivery to the lungs) [80], antibiotics (Ciprofloxacin-conjugated AuNP for extended drug release) [81], protein (Che a-2 allergen delivery to DCs by surface aptamers) [71], nucleic acids (miR_1_-AuNP to ovarian cancer and neuroblastoma) [82], isotopes (^18^F-fluorobenzoate as a PET imaging agent) [83], and glucose- and CXCR-4-coated AuNPs to target human cancer cells to enhance radiosensitization [84,85]. Moreover, AuNPs offer therapeutic possibilities that gold salts lack, specifically, enhancing radiotherapy through the preferential absorption of X-rays by high-atomic-number gold NPs [72,86,87]. While AuNPs are biocompatible in nature owing to their inert and nontoxic nature, their cytotoxicity is dependent on factors like size, shape, surface properties, chemical composition, environmental conditions, and production methods, which warrant further evaluation [88,89]. Notably, the cytotoxic effects of AuNPs are considerably lower, if not absent, compared to other NPs like silver NPs [90].

The nanometer size of such materials plays a crucial role in their biological effects, even for seemingly inert substances like gold. Generally, NPs like AuNPs exhibit low toxicity, similar to other inorganic NPs such as magnetic NPs. At high doses, many nanotherapeutics may become toxic, as demonstrated for titanium dioxide NPs [91]. In vivo studies have indicated the non-toxic effects of 12.5 nm AuNPs in diverse organ systems such as the lungs, liver, spleen, kidneys, and brain [92]. In vitro studies report a size-dependent toxicity of AuNPs, particularly for 1.4 nm particles and not for larger (~15 nm) or smaller (~0.8 nm) particles [93]. Furthermore, minimal sub-lethal toxic effects were observed for different-sized AuNPs (3 nm, 10 nm, 50 nm, and 100 nm) in zebrafish embryos [94]. These results suggest that the tissue distribution and accumulation of AuNPs are size-dependent, with smaller NPs exhibiting the most extensive organ distribution [95,96]. For example, 10 nm AuNPs distribute and accumulate in various organ systems, including the blood, liver, spleen, lung, kidney, thymus, testis, heart, and brain, whereas larger particles (50 nm, 100 nm, and 250 nm) are only detected in the blood and accumulate mainly in the liver and spleen [96]. Additionally, the size and shape of AuNPs impact cellular uptake and biodistribution, resulting in varying accumulations in different organs [95,97]. Therefore, these factors need careful consideration when designing nanomaterials.

Studies have shown that small AuNPs (~4–13 nm) circulate in the bloodstream for 24 h before being cleared within 7 days, as compared to large AuNPs (~100 nm) that are cleared within 24 h. AuNPs of all sizes tend to exhibit a resurgence in the bloodstream at 3 months, correlating with organ levels. Small AuNP accumulation peaks in the liver and spleen at 7 days and in the mesenteric lymph nodes at 1 month, with sustained high levels until about 6 months, indicating a slow elimination process. In contrast, large AuNPs are rapidly taken up by the liver, spleen, and mesenteric lymph nodes within about 30 min, with a less prominent elimination phase [98].

## 5. Interactions Between AuNPs and Immune Cells: A Focus on Immunomodulation

**Figure 3:**
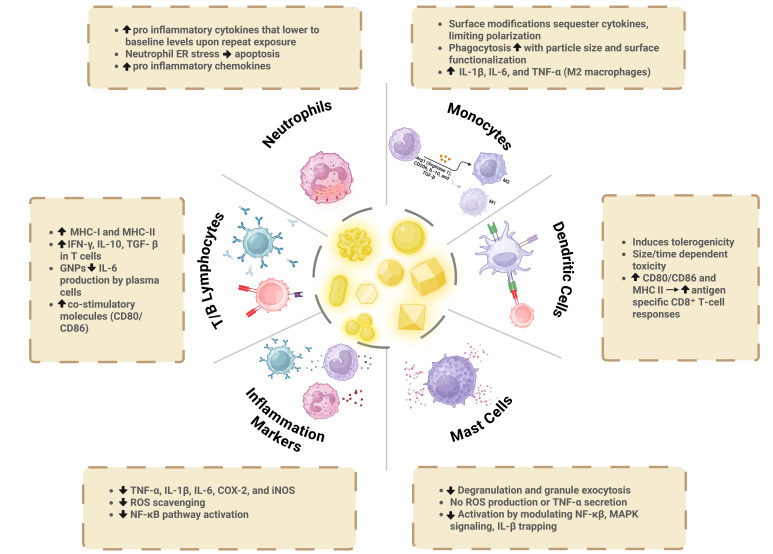
Key effects of AuNPs on innate and adaptive immune cells. AuNPs induce neutrophil apoptosis and downregulate pro-inflammatory cytokine release to curtail acute inflammation. In dendritic cells, AuNPs induce a tolerogenic response to diminish antigen presentation. Upregulation of antigen presenting and co-stimulator molecules on dendritic cells and T cells can be exploited to fabricate effective vaccine adjuvants.

AuNPs have found extensive applications in diagnostics, imaging, targeted drug delivery, and therapy. The effectiveness of AuNPs relies on their ability to target specific cells and be internalized, a process influenced by factors such as surface chemistry (including charge and ligand organization patterns), charge, size, and shape. As these NPs enter and accumulate within cells, they can potentially interfere with cellular function. This becomes particularly significant when considered in the context of the immune system, as some immune cells actively capture extracellular materials and regulate the immune response by activating inflammatory signaling, which stimulates and guides antigen presentation, ultimately leading to immune activation. In this section, we will explore the effects of AuNPs on both innate immune cells (such as DCs, mast cells, and neutrophils) and adaptive immune cells (B and T cells), focusing on their cellular uptake characteristics and subsequent immune response (Figure 3).

Understanding the NP–immune system interaction is crucial to optimizing and developing therapeutic treatments based on AuNPs. The innate immune system serves as the initial line of defense against foreign pathogens and includes circulating and tissue-resident APCs like macrophages and DCs. It is believed that professional phagocytic cells (e.g., DCs, mast cells, monocytes/macrophages, and neutrophils) are the first cells that AuNPs encounter upon entry into the mammalian organism, leading to various physiological responses. Moreover, the higher accumulation of AuNPs in macrophages and DCs due to their phagocytic capacity positions them as suitable candidates for assessing the toxic effects of AuNPs. In the case of high AuNP toxicity, impacts could significantly alter cellular functions due to the ingestion of a large volume of these particles.

On the other hand, the greater accumulation of AuNPs in DCs makes them ideal target cells for specific therapies, particularly immunotherapies, as they enhance the presentation of antigens to the immune system. While numerous studies on the toxicity of AuNPs have indicated that their accumulation in cells induces little to no cytotoxicity [99,100,101], the functional effects of AuNP exposure to immune system cells (known as immunotoxicity) remain relatively unexplored. Later sections of this review elucidate the impact of AuNPs on the metabolism and functions of innate and adaptive immune cells, suggesting that reprogramming cell metabolism with AuNPs could offer a novel therapeutic approach for treating inflammatory diseases. In this section, we will delve into the immunotoxic effects of AuNPs, elucidating the immune response mediated by DCs, macrophages, and other immune cells, with respect to various AuNP parameters such as particle properties like size, dose, shape, and surface functionalization.

### 5.1. Influence of Size and Dosage

Previous studies have established that the size of AuNPs significantly influences their internalization, accumulation, and toxicity. In this context, Shukla et al. [102] conducted seminal studies to investigate the uptake of 3 nm diameter AuNPs by macrophages. Their findings revealed that small AuNPs were taken up by macrophages through pinocytosis. Importantly, their data indicated that these AuNPs were noncytotoxic, biocompatible, and nonimmunogenic. They did not trigger the generation of reactive oxygen species (ROS) or induce pro-inflammatory cytokines like IL-1β and TNF-α. Another study by Yen et al. reported that the administration of AuNPs, particularly those with smaller diameters, led to a decrease in the number of circulating macrophages and an increase in their size, which was accompanied by the upregulation of IL-1β, IL-6, and TNF-α cytokine expression. AuNPs exhibited cytotoxicity and elicited varying immunological responses depending on their size and concentration. Interestingly, the immunological response and cytotoxicity induced by AuNPs were more pronounced when compared to silver nanoparticles (AgNPs). The researchers noted that smaller AuNPs with a negative charge were more likely to adsorb serum proteins on their surface, allowing them to enter cells through endocytosis. This led to a heightened immunological reaction and cytotoxicity compared to AgNPs [103].

The influence of AuNPs on the functions of splenocytes was reported to be both dose- and time-dependent, with differing doses resulting in distinct effects. The lowest dose exhibited a pro-inflammatory effect, stimulating the synthesis of pro-inflammatory cytokines (IL-1β, IL-6, and TNF-α) [104]. However, various studies have produced contrasting results regarding the effects of AuNPs on inflammation. While some studies suggested that AuNPs could attenuate the inflammatory effects of IL-1β or LPS, others observed no impact or even an upregulation of the inflammatory response in macrophages. The size of NPs significantly impacts their interactions with biomolecules within biological systems and their bioactivities. For instance, the binding affinity of Herceptin to its receptor is enhanced over 20 times when loaded onto a 10 nm NP. This affinity further increases ~30 times when a larger NP (70 nm) is employed [105].

In general, AuNPs with a diameter of <5 nm tend to be rapidly cleared through renal filtration [106], and the minimum size to evade renal filtration is ~10 nm [107]. NPs of 10–20 nm sizes are swiftly eliminated through hepatic diffusion [108,109]. Conversely, particles with a diameter exceeding 200 nm are filtered through the splenic sinusoids, recognized, and subsequently cleared by resident macrophages [108,110]. However, nanomaterials falling between the sizes of 20 and 200 nm circulate systemically for extended periods [108,109]. AuNPs ranging from 10 to 22 nm have been proposed as the size threshold for triggering potent immune responses and T cell activation. Consequently, NP size plays an essential role in the design and development of NP-based vaccines [111].

In a study conducted by Abdelmegid et al., 5 nm AuNPs were examined in an ulcerative colitis (UC) mouse model to assess their therapeutic potential. These AuNPs exhibited potent anti-inflammatory effects by downregulating the expression of the pro-inflammatory cytokine IL-17 and decreasing immune-histochemical reactions in the colon. Furthermore, they demonstrated antioxidant properties, leading to a significant reduction in tissue malondialdehyde (MDA) levels. This study suggests that AuNPs could be employed as a novel therapeutic approach for the treatment of UC [112]. Khan et al.’s research emphasized the significant role that AuNP size plays in the immune response. Their findings revealed that while 5 nm AuNPs induced a pro-inflammatory cascade, characterized by increased expressions of IL-6 and IL-1β genes in the mouse brain, larger-sized AuNPs (20 and 50 nm) did not alter the expression of these pro-inflammatory cytokines in the mouse brain, even after repeated dosing [113].

A study by Ni et al. demonstrated that the impact of AuNPs on the regulation of inflammatory responses and the modulation of macrophage polarization was dependent on their size, particularly for AuNPs with sizes of 5, 13, and 45 nm. Their results indicated that AuNPs, especially those with a diameter of 45 nm, not only significantly inhibited LPS-induced M1-polarization and subsequent inflammatory responses—including elevated expressions of CD86, IL-6, nitric oxide synthase (NOS), and TNF-α—but also concurrently upregulated the expression of M2 polarization markers such as Arg1 (Arginase 1), CD206, IL-10, and TGF-β levels. Notably, the 45 nm diameter AuNPs had the most significant impact on macrophage transformation from the “inflammatory M1” to the “regulatory M2” phenotype [114].

In another study, Kirdaite et al. demonstrated that AuNPs with sizes of 13 and 50 nm significantly reduced the inflammatory infiltration of macrophages, leukocytes, and lymphocytes in a size-dependent manner in collagen-induced arthritis (CIA) models [115]. They reported that 50 nm AuNPs were more effective at suppressing inflammation than 13 nm AuNPs, particularly during the early stages of arthritis. This was attributed to the 50 nm AuNPs being internalized more rapidly and in greater numbers compared to other sizes [116]. Additionally, both 13 and 50 nm AuNPs exhibited significant antioxidant properties and enhanced cellular catalase activity without adversely affecting hematological indices or internal organs. In another study by Khan et al., it was observed that treatment with AuNPs led to a considerable downregulation of several pro-inflammatory cytokine mRNA expressions—namely, IL-1β, IL-6, TNF-*α*, NF-κB, COX-2 (cyclooxygenase-2), and iNOS (inducible nitric oxide synthase)—in CIA rat models [117].

Recently, Tomić et al. demonstrated that 10 nm AuNPs can inhibit the LPS-induced production of IL-12p70 by DCs and promote Th2 polarization [88]. Alternately, 50 nm AuNPs were noted to promote Th17 polarization [88]. Another study conducted by Khan et al. reported that both 10 nm and 50 nm AuNPs drastically enhanced the gene expression of pro-inflammatory cytokines (IL-1β, IL-6, and TNF-*α*) in rat hepatocytes after one day of exposure. However, with sub-chronic treatment, these inflammatory responses subsided by the fifth day. Notably, the 50 nm AuNPs induced more severe inflammation compared to their smaller counterparts, suggesting a size-dependent response [118].

Research has indicated that 50 nm AuNPs are particularly efficient in cellular internalization, and spherical-shaped AuNPs tend to have a higher cellular uptake compared to other NP shapes [97]. It has also been observed that smaller AuNPs, those with a diameter of less than 10 nm, can induce systemic toxicity in diverse organs, with the level of toxicity inversely correlating to AuNP size [119]. Moreover, various studies have provided evidence that AuNPs with sizes ranging from 30 to 50 nm exhibit efficient uptake into a variety of mammalian cells. A possible explanation for this observation is that at these sizes, NPs can effectively interact with cellular membranes to form vesicles that mediate internalization [120]. Another study by Zhang et al., which employed 60 nm AuNPs, yielded similar findings in the same cell culture, with no significant increase in the generation of pro-inflammatory mediators [121].

Additionally, Kingston et al. conducted research demonstrating that 50 nm diameter AuNPs did not impact macrophage viability or inflammatory cytokine production on their own. However, they did significantly decrease the levels of ROS and inflammatory cytokine generation and dampen the Th17 response triggered by LPS. Notably, the AuNPs reduced elevated levels of IL-17 and TNF-α in a dose-dependent manner. These results suggest that AuNPs do not exhibit cytotoxicity toward immune cells, but may alter their responses to infection or inflammation by altering the cytokine balance [122]. In parallel, a previous study showed that 15 nm diameter AuNPs reduced elevated IL-17 levels in allergic inflammatory conditions [71]. Notably, 5 nm diameter AuNPs completely inhibited the inflammation induced by IL-1β, whereas 15 nm diameter AuNPs were less effective, and 35 nm diameter AuNPs did not exhibit any significant effect. This marks the first report indicating that citrate-stabilized AuNPs can diminish and/or inhibit the IL-1β-induced inflammatory cascade in a size-dependent manner [123]. Further, Chen et al. demonstrated the anti-inflammatory potential of AuNPs in a rat model of hepatic injury, with a dose-dependent increase in the secretion of anti-inflammatory cytokines (IL-10) from macrophages [124].

Apart from size, AuNPs also exert a dose-dependent effect. In murine models, the oral administration of gold colloid at varying doses (0.25, 2.5, and 25 ppm) for prolonged durations (4 weeks) resulted in distinct, dose-dependent effects on splenocytes. The lowest dose (0.25 ppm) induced a pro-inflammatory response. The medium dose (2.5 ppm) reduced cytokine synthesis (macrophages) while enhancing lymphocyte proliferation, suggesting an immunoregulatory role. The low and high doses induced pro-inflammatory cytokine production, and prolonged (28 days) high-dose exposure led to immunotoxicity, probably due to immune system exhaustion as a result of chronic exposure [125]. In a study by Malaczewska et al., AuNPs up to 10 ppm in mouse splenocytes were found to be non-toxic. Low concentrations (~0.3 ppm) stimulated T cell proliferation and enhanced LPS-induced splenocyte proliferation. Higher doses (5–10 ppm) tended to suppress IL-2 production and slightly reduced cell viability [126].

In a separate study, 50 nm AuNPs exerted a dose-dependent reduction in ROS and TNF-α levels in RAW 264.7 macrophages, with significant effects observed at 25 and 50 μg/mL, with the 50 μg/mL group exhibiting the strongest anti-inflammatory response and no impact on cell viability across treatment concentrations [122]. Rizwan H et al. employed AuNPs to reduce CCl_4_-induced liver toxicity and found that AuNPs administered orally for 4 weeks reduced serum AST and ALT profiles in tetrachloromethane-injured rats. The effect was dose-dependent, with the highest dose (10 ppm) showing the maximum hepatoprotective and anti-inflammatory effects, partly via increased IL-10 levels [124]. In high-glucose-exposed macrophages, AuNPs exerted anti-oxidative effects at non-toxic doses (1–20 nM); increasing doses progressively restored antioxidant capacity and inhibited NF-κB signaling. Doses of >20 nM were reported to be toxic [127].

Summarily, the size and dosage of AuNPs are vital to determine their immunological effects and cytotoxicity. It is broadly observed that smaller AuNPs (particularly with sizes of <10–15 nm) are avidly taken up by immune cells, and they are more likely to drive pro-inflammatory responses(particularly in monocyte/macrophages). Larger AuNPs (20–50 nm) tend to exert anti-inflammatory effects. However, these biological effects are not universal and depend on other factors such as surface chemistry, concentration, and the immune cell content. In DCs and T cells, larger AuNPs appear to be more immunogenic, driving DC maturation, T cell activation, and cytokine release.

### 5.2. Impact of Shape

It has been established that the various physicochemical properties of AuNPs, such as their shape, size, hydrophobicity, and surface characteristics (including coating and charge), play pivotal roles in influencing cellular immune responses. These properties impact cellular uptake and the production of cytokines and ROS, as well as the overall function and cytotoxicity of AuNPs. Previous research has demonstrated that the shape of AuNPs can significantly influence their toxicity, internalization, accumulation, and immune response. Among the varying shapes of AuNPs, nanospheres and nanorods are the most extensively investigated structures for potential biomedical applications due to their well-defined synthesis methods. However, various other structures, including triangular NPs, nanoflowers, nanopyramids, nanoshells, and more, have also been studied. Notably, spherical-shaped AuNPs exhibit higher levels of cellular internalization in contrast to other non-spherical nanostructures [97].

The shape of AuNPs also plays a crucial role in their interaction with macrophages and their subsequent immune responses. For instance, research has shown that gold nanorods (measuring 10 × 45 nm) are engulfed by macrophages to a lesser extent than nanospheres (50 nm) of a similar size and possessing PEGylated surfaces. This reduced phagocytosis efficiency of nanorods can be attributed, in part, to their prolonged circulation in the bloodstream, resulting in diminished accumulation in macrophage-rich organs such as the spleen and liver, and, therefore, higher accumulation in tumor tissues. Additionally, the negative surface charge of gold nanospheres may contribute to their increased uptake [128].

In vitro studies have further explored the impact of aspect ratios on the behavior of PEGylated nanospheres (18 nm) and nanorods (14 × 58 nm and 16 × 110 nm), with aspect ratios of one, four, and seven, respectively, concerning macrophages, tumor cells, and immune responses. Intriguingly, high-aspect-ratio nanorods demonstrate more efficient entry into macrophages but exit more rapidly, resulting in prolonged accumulation within tumor cells compared to low-aspect-ratio and spherical NPs. Macrophages treated with high-aspect-ratio nanorods release higher quantities of pro-inflammatory cytokines (IL-6 and TNF-α), while gold nanospheres induce a weaker immune response [129].

AuNPs’ shape also influences their interactions with cellular membranes. While nano stars (NSTs) disrupt and penetrate lipid bilayers, causing significant membrane damage and increased lipid order, nanospheres (NSs) primarily induce membrane bending without disruption [130].

Xie et al. demonstrated that AuNPs’ shape significantly influences their uptake by macrophages. Gold nanotriangles (NTs) showcased the highest internalization rates, followed by nanorods (NRs) and nano stars (NSTs); while all shapes exploited clathrin-mediated endocytosis, NRs also engaged caveolae-mediated pathways. NTs depended heavily on dynamin and cytoskeletal involvement. The increased bending energy of NSTs probably hindered uptake due to increased membrane bending energy [131]. In RAW 264.7 macrophages, gold NSTs (multibranched, ~100 nm) have been shown to result in pronounced pro-inflammatory activation (significant TNF-α secretion and ROS generation—levels comparable to LPS stimulation) in contrast to spherical core–shell Fe_3_O_4_@Au nanoparticles (~34 nm). Further, while NSs induced cell detachment and rounding, NSTs resulted in enlargement and immune activation [132].

Further, in a study by Vandebriel RJ et al., PEGylated gold nanorods (not NSs or NSTs) robustly activated the NLRP3 inflammasome in macrophages, highlighting a shape-specific inflammatory response. This nanorod-induced inflammasome activation was associated with distinct transcriptomic alterations (the suppression of cholesterol biosynthesis, augmented oxidative phosphorylation, and activation of PON2—a key oxidative stress regulation), emphasizing the immunomodulatory influence of nanoparticle anisotropy and surface chemistry [133].

To summarize, AuNPs’ shape influences their cellular uptake and biodistribution. Spherical AuNPs are internalized more efficiently into macrophages, and cellular interaction with anisotropic shapes is complex. High-aspect-ratio gold NRs and NSTs have been shown to trigger inflammatory responses. Such distinct shape-dependent cellular effects highlight the importance of morphology in guiding nanomaterial design for safe and effective biomedical applications.

### 5.3. Significance of Surface Functionalization

The core composition and surface coating of NPs represent another critical factor that exerts a significant influence on immune responses [134]. When AuNPs interact with biological systems, they encounter diverse proteins and metabolites. These interactions trigger alterations in the physicochemical properties of AuNPs, such as altering their size, surface chemistry, and charge, and may result in aggregation or disassembly. These changes, in turn, profoundly impact processes like cellular uptake, cytotoxicity, and immune responses. The adsorption of serum proteins onto the AuNPs surface is referred to as the “protein corona” [101].

Notably, Bastús et al. discovered that bare 10 nm AuNPs did not influence the production of nitric oxide (NO) and other pro-inflammatory cytokines. However, when AuNPs were surface-modified with peptides like amyloid growth inhibitory peptide (AGIP) or sweet arrow peptide (SAP), they were recognized by TLR4 receptors, leading to prolific entry into macrophages. Subsequently, these modified AuNPs inhibited macrophage proliferation, iNOS synthesis, and the production of pro-inflammatory cytokines (IL-1β, IL-6, and TNF-α) [135]. In contrast, Staroverov et al. demonstrated that both naïve 15 nm diameter AuNPs and their antigen-coated conjugates could influx into rat peritoneal macrophages, stimulate the respiratory activity of macrophages, enhance mitochondrial enzyme activity, and augment IL-1β, IL-6, and IFN-γ production. They proposed that this stimulation may be crucial in determining the extent of adjuvant potential of colloidal gold [136,137]. These studies collectively emphasize that AuNP surface chemistry can significantly impact macrophage immune response post-uptake [135,136,137].

The activation of macrophages by AuNPs, as evidenced by multiple researchers, holds significant potential for the development of novel vaccine adjuvants. In another in vitro study by Wei et al. [138], it was demonstrated that 15 and 30 nm AuNPs, conjugated to CpG (cytosine–phosphate–guanosine) oligodeoxynucleotides, exhibited a remarkable capacity to enhance NP internalization by macrophages. Furthermore, this resulted in an elevated production of pro-inflammatory cytokines such as TNF-α and IL-6, particularly by the 15 nm conjugates. The researchers reported that AuNP–CpG conjugates exerted a more potent immunostimulatory effect compared to CpG alone at equivalent concentrations. Consequently, these conjugates showed promise in effectively addressing the primary challenges associated with intracellular delivery of oligodeoxynucleotides, highlighting their potential as a valuable therapeutic tool in animal studies [138].

Furthermore, Sumbayev et al. reported that citrate-stabilized AuNPs failed to activate the inflammasome pathway in the THP-1 cell line, in addition to downregulating the inflammatory process by selective inhibition of the IL-1β-dependent pathway. They demonstrated that the anti-inflammatory activity of AuNPs can mainly be attributed to their interference with the transmission of inflammatory signaling through extracellular interactions with IL-1β. They professed that AuNPs aggregated around extracellular IL-1β, obstructing its binding to cellular receptors. The 5 nm AuNPs exhibited maximal inhibitory effects, owing to their high specific surface area, which probably enhances their affinity to IL-1β [123]. In contrast, another study showed that negatively charged poly(acrylic acid)-conjugated AuNPs exerted a pro-inflammatory response in the THP-1 cell line and promoted integrin activation [139]. The core and surface coating of NPs can affect their properties and effects, and similar NPs with different surface chemistries can potentially display altered interactions with key signaling proteins [135], leading to drastically different interactions with the main signaling proteins. While citrate-stabilized AuNPs inhibited inflammatory processes, the poly(acrylic acid)-coated AuNPs induced pro-inflammatory effects.

The surface charge of AuNPs is crucial in determining their solubility, hydrophobicity, molecular binding, and interactions with various cell types [140]. It has been reported that positively charged NPs can induce stronger inflammatory responses because cationic NPs more effectively bind to negatively charged cell membranes, enhancing phagocytosis compared to neutral or anionic NPs [141]. Additionally, research has revealed that both positively and negatively charged AuNPs can be observed in cytosol and lysosomes, but not in mitochondria or the nucleus [142]. Positively charged 15 nm AuNPs entered cells and significantly influenced cellular responses by modulating the expression of both anti-inflammatory (TGF-β) and pro-inflammatory (IL-1β) cytokines, whereas negatively charged AuNPs mainly stimulated the expression of TNF-α. Cationic AuNPs, regardless of size, were preferentially internalized and underwent aggregation in the macrophage cell line (phorbol-12-myristate-13-acetate-differentiated U937-macrophages) for extended durations compared to AuNPs with other charges (zwitterionic, anionic, and PEGylated AuNPs). The intracellular agglomeration seemed to delay their exocytosis. In contrast, PEGylated AuNPs migrated within the cytosol as individual particles and consequently had the highest rate of exocytosis. The anionic and zwitterionic-charged AuNPs demonstrated intermediate exocytosis rates. Interestingly, the exocytosis patterns were not influenced by size and remained similar in the size range of 10–40 nm, indicating that the surface charge of AuNPs trumps size as the major determinant of the cellular excretion of sub-40 nm AuNPs [143]. This underscores the importance of considering the surface charge of NPs when designing and using AuNPs for diverse in-human applications, such as for drug delivery, immunomodulation, and vaccination [144].

Functionalizing AuNPs with peptides has emerged as a promising strategy to shield biomolecules against enzymatic hydrolysis while in circulation, thereby enhancing passive targeting, tissue penetration, and improving the uptake of biomolecules by macrophages [145]. This strategy also plays a crucial role in modulating the immune response, more so for proteins that are otherwise unrecognizable by macrophages. Some studies have demonstrated that modifying the surface of AuNPs with peptides and proteins holds promise for activating and polarizing macrophages to an anti-tumor phenotype. This approach has found application in vaccination experiments. For instance, ovalbumin (OVA)-decorated AuNPs were more efficiently taken up by the RAW264.7 macrophage cell line compared to PEG (polyethylene glycol)-modified AuNPs. These OVA-decorated AuNPs inhibited tumor growth by inducing higher levels of inflammatory factors (IL-1β, IL-6, and TNF-α) by activated macrophages [146]. Mucin 1 (MUC1), a membrane-tethered glycoprotein expressed in glandular epithelial tumors of gastrointestinal origin, has been targeted for tumor immunotherapy. Modified MUC1 protein-functionalized AuNPs were found to be potent macrophage activators, promoting the release of IL-6, IL-10, IL-12, and TNF-α from peritoneal macrophages and driving predominant M1 polarization, highlighting their potential as a potent tumor vaccine [147].

Furthermore, AuNPs can impact cytokine production based on their surface charge and differing types of surface-bound peptides in a cell-type-dependent manner. Bartneck et al. have reported that while the surface charge of AuNPs primarily influences uptake, the surface-coupled peptide sequences alter cellular functions, modifying the activation profile of DCs and modulating cytokine release in both DCs and macrophages in a cell-specific fashion. AuNPs can also bind to diverse biomolecules and expose smaller molecules to the immune system, those traditionally unrecognized by macrophages. Therefore, peptide-functionalized AuNPs, known to activate macrophages, hold promise for application as vaccine adjuvants. Moreover, AuNPs can be conjugated with adjuvants, adaptor proteins, or cofactors to effectively enhance immune responses [148].

Briefly, data shows that AuNPs with negatively charged citrate and poly(acrylic) coatings exert pro-inflammatory effects. Further, charge also governs cellular internalization and exocytosis. Consequent to their prolonged intracellular retention, cationic AuNPs can also trigger potent immune activation. The peptide and antigen functionalization of AuNPs (with OVA and CpG) can aid in targeting immune cells and triggering pro-inflammatory responses.

## 6. Anti-Inflammatory Effects of AuNPs on Innate Immune Cells

### 6.1. Neutrophils

Neutrophils constitute the majority of white blood cells, comprising over 65% of circulating leukocytes. They swiftly migrate to sites of inflammation, initiating the host’s response. Notably, neutrophils play a vital role in phagocytizing foreign particles and pathogens, making them frequent points of contact for administered NPs [149]. Despite their pivotal role in inflammation and NP phagocytosis, there has been limited investigation into how AuNPs affect neutrophil function, and the mechanisms underlying their impact on neutrophil biology remain a poorly explored area of research.

Research has indicated a size-dependent effect on NP uptake by neutrophils, with larger NPs (ranging from 20 nm to 200 nm) demonstrating more efficient internalization than smaller ones [150]. Several types of NPs, including gold, silver, and cationic lipids, have been linked to triggering NETosis, a process in which neutrophils release neutrophil extracellular traps (NETs). Unlike macrophages and monocytes, which engulf NPs, neutrophils are observed to trap AuNPs in NETs [151,152,153]. A study by Bartneck et al. explored the interaction of AuNPs ranging from 15 to 50 nm in diameter with neutrophils, revealing that AuNPs were predominantly located within neutrophils within just 15 min of immune cell exposure, as opposed to macrophages and monocytes. NETs seemed to act as a physical barrier for NPs, and while particle shape (rod-shaped or spherical) did not significantly affect trapping, positively charged functionalized AuNPs were more frequently trapped by NETs, possibly due to the net negative charge of DNA, a major component of NETs [152].

Another study delved into how 60 nm diameter AuNPs induced free radical generation, which is involved in NET formation [154]. The uptake of AuNPs has been associated with triggering endoplasmic reticulum stress and the cleavage of cytoskeletal proteins in human neutrophils, ultimately cascading into apoptosis (up to 3-fold compared to untreated controls) [155]. Despite both 20 nm and 70 nm diameter AuNPs (both negatively charged) being taken up through different pathways—cytosolic vacuolar internalization and cell membrane localization, respectively—both sizes triggered ER stress-induced apoptosis. Therefore, AuNPs can serve as novel agents inducing proapoptotic effects in neutrophils, exhibiting toxicity towards them [155].

In line with these findings, another study by the same research group suggested that both positively and negatively charged AuNPs were ingested by human neutrophils, enhancing spontaneous apoptosis and increasing gelatinase activity. Additionally, these AuNPs prompted the production of at least two inflammatory chemokines (GRO-α and IL-8), correlating with the induction of neutrophilic inflammation in vivo, albeit to varying degrees. Notably, positively charged AuNPs exhibited a higher pro-inflammatory potential in contrast to negatively charged AuNPs, although the underlying reasons for this difference remain unclear and require further investigation [156].

In a study by Khan et al., the effects of AuNPs on the innate pro-inflammatory cytokines’ (e.g., IL-1β, IL-6, and TNF-α) mRNA expression were evaluated in a rat model. They observed that AuNPs with sizes of 10 and 50 nm significantly increased pro-inflammatory cytokine release (2.46-fold and 8.26-fold increase in IL-6 by 10 and 50 nm AuNPs, respectively; 6.3-fold and 22.65-fold increase in TNF-α by 10 nm and 50 nm AuNPs, respectively) on day 1, which gradually decreased by day 5, suggesting a transient acute-phase response followed by exhaustion to near baseline upon repeated exposure [157]. The exact underlying mechanisms of AuNP effects on neutrophils remain unclear, necessitating further research in this area before definitive conclusions can be drawn.

### 6.2. Monocytes/Macrophages

Following neutrophils, monocytes and macrophages become the predominant cells at inflammation sites. These cells, which serve as professional APCs, are particularly affected by the presence of NPs, including AuNPs. In this section, we will explore several influencing factors associated with AuNPs that impact their interaction with macrophages (Table 1). Although the precise mechanism is ambiguous, the ability of AuNPs to engage with macrophages and influence macrophage polarization is closely tied to factors such as the AuNP shape, size, and surface properties, and also the macrophage phenotypes.

Numerous studies have revealed that the macrophage uptake of spherical AuNPs and their subsequent immune response vary not only based on particle size, but also on whether the particles are unmodified or have undergone surface modifications. In the case of unmodified gold colloids protected by trisodium citrate, small NPs exhibit higher macrophage uptake and exert a more significant anti-inflammatory effect compared to larger ones. It is important to note that the interaction between AuNPs and macrophages is dynamic, with both entities influencing each other. The physicochemical parameters of AuNPs (shape, size, surface charge, and surface chemistry) clearly impact macrophage endocytosis, the exocytosis of NPs, and their self-polarization. Conversely, macrophage phenotypes can also alter the uptake and efflux of AuNPs [50,158,159,160].

Based on the results mentioned above, it is apparent that unmodified AuNPs, regardless of size, are generally noncytotoxic, biocompatible, and nonimmunogenic to macrophages. However, both small, unmodified gold colloids and larger AuNPs adorned with biocompatible molecules effectively limit macrophage polarization by binding to cytokines and other related biomolecules. It is also worth noting that variations in AuNP synthesis protocols, the protective agents employed to coat AuNPs, and the macrophage state can yield different outcomes in various studies. Therefore, the size effect of AuNPs on their interaction with macrophages should be interpreted within the context of specific experimental conditions. In a study by Taratummarat et al., AuNPs (21.3  ±  0.7 nm) were administered to mice in a model of bacterial sepsis as a potential adjuvant to antibiotics. The results demonstrated that AuNPs significantly decreased pro-inflammatory polarization (M1: CD86^+ve^ in F4/80^+ve^) while accentuating the anti-inflammatory polarization of macrophages (M2: CD206^+ve^ in F4/80^+ve^) in the splenic tissue. This shift was also accompanied by a decrease in IL-1β, IL-6, iNOS, Nur77, and TNF-α, as well as an increase in Arg1 and PPARγ levels [161].

Another study indicated that among various PEG-modified AuNPs with sizes of 15, 60, and 100 nm and an equal surface modification density, larger NPs were more susceptible to phagocytosis by all macrophage phenotypes (including M1, M2, M2a, M2b, and M2c, derived from human monocytes). Notably, M2 macrophages exhibited a greater tendency to uptake PEGylated AuNPs, with the following phagocytic ability ranking: M2c > M2 > M2b > M2a > M0 > M1. Also noted was the observation that both pre- and post-treatment with PEGylated AuNPs inhibited the polarization of LPS-stimulated macrophages, especially those treated with larger AuNPs, particularly PEGylated AuNPs with a size of 100 nm [162]. In another study, a trend was observed among functionalized AuNPs coated with PEG, chicken protein, or ovalbumin (OVA) in sizes of 12, 35, and 60 nm, where larger particles or those conjugated with OVA were more readily phagocytosed by macrophages. This also resulted in significantly higher production and secretion of IL-1β, IL-6, and TNF-α. Conversely, PEG-coated AuNPs failed to trigger any significant inflammatory response, particularly by particles sized >35 nm. Summarily, smaller AuNPs triggered significant inflammatory reactions, regardless of the differing surface coating used [146]. These findings underscore the impact of NP shape on immune responses in macrophages, which may promote the lysosomal exocytosis of NPs [163].

### 6.3. Dendritic Cells

Understanding the NP–immune system interaction is crucial to optimizing and developing AuNPs for therapeutic applications. AuNPs have been extensively researched as potential carriers for delivering antigens to DCs. DCs, as professional APCs, are key in initiating, developing, and activating T cells, and are, thus, an indispensable bridge between the innate and adaptive immune systems. Studies, particularly in the context of antigens associated with viruses and tumors, have demonstrated successful targeted antigen delivery to DCs using AuNP carriers. However, it is important to note that AuNPs can exert immunostimulatory effects on DCs and macrophages even in the absence of antigens. It has been observed that AuNPs do not significantly alter the fundamental functions of macrophages and DCs, but their exposure can differentially affect the responses of these immune cells to subsequent antigen stimulation, including the secretion of inflammatory mediators such as cytokines (TNF-α, IL-6, and IL-1β), chemokines (e.g., MCP-1), and ROS [100].

Investigations into the interactions of AuNPs on DCs and subsequent effects are of paramount importance due to the critical role that DCs play in initiating both innate and adaptive immune responses. Focusing on human DCs is particularly essential, given the therapeutic potential of AuNPs in human diseases. Multiple pathways regulate the anti-inflammatory function of DCs. For instance, increased IL-27 signaling and CD39 cell surface expression in DCs limit T cell-mediated inflammation. The maturation of DCs can be tracked by detecting specific surface markers, including CD80, CD83, CD86, and HLA-DR, upon antigen presentation to DCs. CD80 and CD86 are indicative of mature DCs, while CD83 is considered a marker of activation. DCs are well-established regarding their capacity to prime T cell activity and trigger a robust T cell response. Consequently, targeting these immune cells represents a highly effective strategy to develop immunotherapies and vaccines [164].

#### 6.3.1. Impact of Surface Functionalization on DC Function

AuNPs possess several favorable characteristics, including facile and stable surface functionalization with targeting agents or cell-penetrating peptides and the controlled release of therapeutic agents. These attributes position them as potential tools for targeting DCs to enhance antigen uptake, achieve intracellular targeting, and improve penetration through tissue and mucosal barriers. Studies have underscored the significance of the physicochemical properties of AuNPs in modulating DC functions and subsequent immunological effects. Surface chemistry, encompassing factors like charge, AuNP modifications, protein corona formation, and ligand organization patterns, is pivotal in determining the internalization of AuNPs by DCs. For instance, a positively charged surface on AuNPs enhances their uptake efficiency by human monocyte-derived DCs [165]. In this regard, zwitterionic ligand-stabilized AuNPs have been recorded to induce DC maturation and elicit a tolerogenic response characterized by a Th1/Treg pattern, particularly in the absence of NK and cytotoxic T cells. Interestingly, the protein corona surrounding NPs is another critical parameter influencing the immune response, with zwitterionic ligand-conjugated AuNPs exhibiting more effective uptake compared to PEGylated ones [166].

Conversely, a PEG coating of AuNPs tends to conceal them, evading cellular uptake mechanisms and resulting in lower uptake by DCs [167]. Additionally PEG and polyvinyl alcohol-coated AuNPs can be modified to impart a negative surface charge (-CO_2_H), permitting the highest cellular uptake by DCs, suggesting the importance of a combined approach involving both surface charge and surface modification strategies to modulate cellular uptake in DCs [165]. The functionalization of AuNPs with targeting agents specific to DC surface receptors can further increase cellular uptake while reducing systemic toxicity [71,168,169]. For example, DC-SIGN-targeted AuNPs are being explored as neutral carriers, as these demonstrate the ability to induce DC-SIGN internalization, albeit without triggering DC maturation or IL-10 induction. This suggests their potential application as antigen delivery devices and targeted imaging tools [168].

It is worth noting that coating AuNPs with NH_2_-PVA significantly increases internalization (a threefold increase) in DCs compared to an NH_2_–PEG coating [167]. However, it is crucial to maintain the proper surface functionalization of AuNPs, as polymer-coated AuNPs with positive charges have been shown to significantly reduce cell viability [165], emphasizing the importance of meticulous surface modification to ensure safe application in humans. Furthermore, the appropriate synthesis of AuNPs using purified chemical reagents, such as free citrate and cetyltrimethylammonium bromide (CTAB), as well as their complete removal, is essential to avoid cellular toxicity [101]. Cumulatively, these findings underscore the significance of the precise synthesis and characterization of AuNPs in minimizing their cytotoxicity in experimental cell lines. To delve specifically into DCs, Fytianos et al. coated AuNPs with PVA functionalized with either carboxylate (CO_2_^−^) or amine (NH_3_^+^) functional groups. Their results demonstrated that AuNP-PVA with NH_3_^+^ induced a superior DC uptake efficiency (30% vs. 5%) compared to CO_2_^−^. Both types did not exert any cytotoxicity [170].

#### 6.3.2. Influence of Size, Dosage, and Time of Exposure of AuNPs on DCs

The toxicity of AuNPs to DCs is significantly influenced by the size of AuNPs and the exposure time. For instance, during a short exposure period (up to 48 h) to AuNPs with sizes ranging from 10 to 80 nm, no toxicity was observed [171]. Conversely, an in vivo study demonstrated that prolonged exposure to AuNPs (up to 21 days) led to size-dependent toxicity. In murine models, smaller AuNPs (8 nm) induced severe toxicity over 21 days, while larger ones (50–100 nm) did not exhibit any toxicity [98]. Interestingly, some studies have reported an inverse correlation between AuNP size and uptake efficiency by DCs [172,173]. In certain cases, smaller-sized AuNPs (<2 nm diameter) were observed to be more readily internalized into cellular compartments through diffusion and other internalization mechanisms [166,174]. Nonetheless, an ideal AuNP size must strike a balance between being large enough to load therapeutic agents as carriers and avoiding elimination before interacting with DCs. Size and surface coating also influence diffusion rate, excretion, and, consequently, the half-life of AuNPs. Although smaller-sized AuNPs (5–15 nm) exhibit wider distribution, medium-sized AuNPs are preferred due to their superior internalization. AuNPs sized within 40–60 nm are well-tolerated and more efficiently internalized by DCs compared to other sizes. For instance, Tomic et al. demonstrated that 50 nm AuNPs were internalized more efficiently by DCs compared to their 10 nm counterparts [88].

Numerous studies have indicated minimal cytotoxicity associated with different sizes of AuNPs on DCs and T cells, regardless of their concentrations and surface functionalization. Bare AuNPs, for instance, do not stimulate the secretion of cytokines and chemokines, such as IL-1β, IL-6, and TNF-α, or the production of NO and ROS by DCs and macrophages, as reported by various studies [99,121,166,175]. Furthermore, Dey et al. reported that while AuNP exposure did not significantly alter the functions of DCs and macrophages, it did affect their responses to subsequent stimulations differently [100]. Another study found that 10 nm AuNPs were not cytotoxic to bone-marrow-derived DCs (BMDCs), even at high concentrations, but did suppress the immune response in murine BMDCs, evidenced by reduced LPS-mediated secretion of IL-12p70 and IL-1β [99].

Interestingly, AuNPs have also been shown to induce immunosuppression in DCs. Fernandez et al. reported that treatment of human DCs with ultra-small gold nanoclusters (~3 nm) generated an immunosuppressive response characterized by increased IL-10 secretion. In essence, data revealed a dose- and time-dependent intracellular accumulation of AuNPs in human DCs, with size and ligand type playing pivotal roles in the efficient cellular uptake and immune stimulation of DCs. Conversely, larger AuNPs (approximately 12 nm) triggered an inflammatory and cytotoxic response involving NK cells in a ligand-independent manner [176]. In human DCs, AuNPs with a diameter of 10 nm inhibited the expression of CD83, CD86, and IL-12p70 when stimulated with LPS. For instance, in a study by Tomić et al., unmodified AuNPs with diameters of 10 and 50 nm did not affect the activation of DCs, as evidenced by CD83, CD86, and MHC-II surface expression, but suppressed the LPS-induced upregulation of CD83, CD86, and MHC-II [88].

Fundamentally, the cellular uptake and internalization of antigens by DCs typically leads to DC maturation and the initiation of T cell responses. However, the uptake and internalization of AuNPs do not universally result in enhanced DC maturation and the activation of T cell-mediated immunity. Several factors, including size, surface functionalization, dosage, and exposure time, can distinctly influence DC maturation, steering them toward tolerogenic or immunogenic subtypes and shaping the fate of T cell responses.

### 6.4. Mast Cells

Mast cells play a pivotal role and are widely recognized for their involvement in hypersensitivity type I reactions (commonly known as allergies). Additionally, they contribute to both innate and acquired immunity. Recent research has shed light on mast cell function in various inflammatory diseases, namely multiple sclerosis, atopic dermatitis, arthritis, and psoriasis. In these conditions, mast cells are activated by non-allergic triggers and are primed to release pro-inflammatory mediators. Notably, mast cells release IL-1, which, in turn, selectively triggers the secretion of other inflammatory cytokines like IL-6 and TNF-α. This activation can occur through the engagement of mast cell receptors such as TLRs and IgE–FcεRI and involves signaling pathways like PI3K, ERK, JNK, NF-κB, and PKC [177].

Interestingly, Huang et al. demonstrated that dinitrophenyl-AuNPs (size ~25.8 nm) could modulate the cross-linking of IgE–FcεRI receptors on the surface of leukemic cells (RBL-2H3 cell line), a cell line known for releasing histamine. This interaction resulted in inhibitory signals [178]. Marquis et al. conducted two studies, indicating that citrated AuNPs can both trigger degranulation and inhibit granule exocytosis from the peritoneal mast cells of mice, with the effect being time-dependent. Initial exposure up to 48 h stimulated degranulation, whereas prolonged exposure up to 72 h led to the inhibition of granule exocytosis [179,180]. In another study by Gutiérrez-Calleja et al., treatment of the HMC-1 cell line with AuNPs resulted in a slightly diminished cell viability during the first 4 h, with no impact on their proliferation. Moreover, their results demonstrated that AuNPs did not stimulate ROS production or TNF-α secretion at any time or concentration. Thus, AuNP internalization into the HMC-1 cell line did not result in any cytotoxicity or trigger the synthesis of pro-inflammatory mediators [181]. AuNPs can also potentially affect mast cell activation through various mechanisms, including the modulation of the NF-κB and MAPK signaling pathways, IL-β trapping, and interference with TLR4/9 receptors. A comprehensive table highlighting the effects of AuNPs on innate immune cells is provided for readers in Table 1.

**Table 1:** The inflammatory and anti-inflammatory effects of AuNPs on innate immune cells.

Immune Cells	AuNPs Properties	Model/Cell Line	Mechanism of Action	Main Effect		Ref.
				Inflammatory	Anti-Inflammatory	
Neutrophils	15–50 nm	Human PBMCs	- Trigger NETosis - ↓Inflammation		✔	[152]
	1–10 nm	Human PBMCs and CD-1 mice	- ↑Proapoptotic effect via IL-8 and GRO-α - ↑IL-6 secretion after AuNP (−) vs. AuNP (+) exposure - ↑Neutrophil activation	✔ (Positive charge)	✔ (Negative charge)	[156]
	20 and 70 nm	Human PBMCs	- ↑ER stress and trigger apoptosis		✔	[155]
Monocytes/Macrophages	10 nm (serum albumin coated)	Splenic macrophages and TAMs	- ↑NADPH oxidase, ROS generation - ↓TNF-α and IL-10; increase IL-12 - Polarize TAMs (M2→M1)	✔		[182]
	5, 15, 20, and 35 nm	C57BL6 mice/THP-1	- ↓NF-κβ and IL-1β-driven inflammation - ↓TNF-α and HIF-1α		✔ (small sizes)	[123]
	nm	CCl4-injured rat liver/RAW 264.7	- Modulating IL-10 secretion - ↓AST and ALT levels		✔	[124]
	50 nm	RAW 264.7	- ↓LPS-triggered IL-17A, TNF-α, IL-1β - ↓ROS		✔	[122]
	10 and 15 nm	RAW 264.7	- ↓NF-κβ and STAT1 activation in LPS-stimulated cells - ↓iNOS mRNA expression and NO production		✔	[183]
	Up to 5 nm	Mice splenocytes	- 2.5 ppm—↓ IL-1β, IL-6, TNF-α; ↑ IL-2 in macrophages - 0.25 and 25 ppm—↑ IL-1β, IL-6, TNF-α; ↓ IL-2	✔	✔	[125]
	20 nm	RAW 264.7	- ↓ NF-κβ activation by modulating the ERK1/2MAPK/Akt/tuberin-mTOR pathways - ↓IL-1β, CCL-8, CX3CL-1, CX3CL-10, ICAM, MMP-2/9, TNF-α expression (dose-dependent)		✔	[127]
	2.81, 5.52, and 38.05 nm	J774 A1	- ↑IL-1β, IL-6, and TNF-α expression (small > large)	✔		[103]
	10–300 nm	RAW 264.7	- ~On IL-6, IL-10, and TNF-α	~	~	[175]
	30–40 nm	Rat hepatocytes and Kupffer cells	- ↑In IL-10 secretion - Controlled IL-6 secretion - ↓In TNF-α secretion		✔	[184]
	20 and 40 nm	RAW 264.7	- ~On the induction of NF-κβ, IL-6 release, or ROS generation	~	~	[185]
	60 nm	RAW 264.7	- Not cytotoxic, nor elicit pro-inflammatory responses and oxidative stress - No additive or synergistic effects on LPS-induced TNF-α and IL-6 production - ~Intracellular ROS	~	~	[121]
	20–50 nm	Primary human monocytes	- ↓Proportion of CD14+/CD16+ monocytes - Significantly ↓CD86 expression - ↓HLA-DR expression - ↓LPS-induced p40 subunit of IL-12 and IL-23 - ↓TNF-α cytokine production		✔	[186]
	Up to 5 nm	Mice splenocytes	- <2.5 ppm: ↑IL-1β; ↓IL-2 and TNF-α - 2.5–5 ppm: No effect - 10 ppm: ↑IL-6 secretion; ↓IL-1β and TNF-α		✔	[126]
	5, 10, 15, 30, and 60 nm	RAW 264.7	- ↓NF-κβ activation via TLR4 signal dampening - ↓IL-6 and TNF-α expression - ↓LPS-triggered iNOS expression, NO production		✔	[187]
	5, 13, 45 nm	RAW 264.7	- ↓LPS-induced M1-related factors (CD86, iNOS, IL-6 and TNF- α) - ↑expression of M2-related factors (Arg1, CD206, IL-10, TGF-β)		✔	[114]
	4, 11, 19, 35, or 45 nm	RAW 264.7	- ↓HMGB-1 and TLR9 binding and suppression of TLR-9 activation - ↓CpG-ODN induced TNF-α production		✔	[188]
	3, 11, 16, 30, and 40 nm	RAW 264.7; SV40; SVEC4-10; C3H10T1/2	- 11 nm AuNPs ↓CCL5 secretion; 16 nm AuNPs increased CCL5 secretion - ↑TNF-α secretion - ~IL-10 secretion - ~IL-6 levels	✔ (Small > large)		[189]
DCs	Unmodified AuNPs of 10 and 50 nm	Human monocyte-derived DCs	- 10 nm AuNPs ↓CD83, CD86, and IL-12p70 in LPS-stimulated cells		✔	[88]
	2 and 12 nm	Human monocyte-derived DCs	- 2 nm: mild Immunosuppression - 12 nm: ↑inflammatory and cytotoxic response	✔ (12 nm)	✔ (2 nm)	[176]
	PVA-NH_2_ and PEG-CO_2_H AuNPs	Human monocyte-derived DCs	- No effect on cytokine secretion by human DCs		✔	[165]
	2 and 12 nm	Human monocyte-derived DCs, PBMCs	- 2 nm: ~DC maturation and lymphocyte proliferation and ↑tolerogenicity - 12 nm: ↑DC maturation	✔ (12 nm)	✔ (2 nm)	[166]
	PVA-coated AuNPs functionalized with DC-SIGN	3D co-culture (epithelial cells, DCs, and macrophages)	- ↑DCs’ uptake efficiency - ~IL-10	~	~	[170]
	2–3 nm DC-SIGN-conjugated AuNPs (*N*-α-fucosyl-β-alanyl amide)	Human monocyte-derived DCs	- ↑DC-SIGN internalization - ~IL-10	~	~	[168]
Mast cells	25.8 nm	RBL-2H3	- Modulate IgE–FcεRI receptor cross-linking		✔	[178]
	Citrated-AuNPs	Murine peritoneal mast cells	- ↓Granule exocytosis		✔	[179,180]
	Citrated-AuNPs	HMC-1	- ↓ROS and TNF-α production - Modulate NF-κβ and MAPKs signaling pathways		✔	[181]

(↓—Decrease ↑—Increase ~—No effect).

## 7. Anti-Inflammatory Effects of AuNPs on Adaptive Immune Cells

The connection between adaptive and innate immune cells is well-established. Innate mediators are crucial in orchestrating adaptive immune responses, including the polarization of T cells into diverse subtypes (Th1, Th2, Th17, Treg, and CTL). Therefore, in the context of different disorders, adaptive immune responses need to shift to mitigate inflammation. For instance, Th1 polarization is desirable in hypersensitivity type I reactions, leading to IgG secretion by B cells. Similarly, in Th1/Th17-related diseases like RA, IBD, and MS, expanding Treg populations can help reduce inflammation. DCs, being essential immune cells, regulate lymphocyte polarization. AuNPs can be employed to deliver antigens to APCs along with adjuvant agents, cytokines, and small activating or inhibitory molecules, thereby influencing APC functions related to T cell activation, differentiation, and inhibition.

### 7.1. T Lymphocytes

#### 7.1.1. Impact of Surface Functionalization on T Cells

AuNPs can potentially bias the differentiation of T cells induced by DCs and enhance the ability of DCs to stimulate their differentiation into Th1, Th2, Th17, and Treg subtypes. Importantly, this effect is closely linked to AuNPs’ surface chemistry. Notably, gold nanorods coated with poly-diallyl dimethylammonium chloride (PDDAC) and polyethyleneimine (PEI) undergo Th2 polarization, whereas those coated with CTAB do not. Furthermore, these modified AuNPs significantly boost both humoral and cellular immunity and promote T-cell proliferation by activating APCs [190]. Gold nanorods have also been shown to enhance DC maturation and downstream immunity; these effects are also influenced by their specific surface chemistry [190,191].

In a study by El-Sayed et al., dextran-modified AuNPs were found to enhance the antigen presentation properties of bone-marrow-derived dendritic cells (BMDCs) in mice. This enhancement was achieved by upregulating the expression of MHC-I, MHC-II, and co-stimulatory molecules (CD40, CD80, and CD86), thereby generating robust Th1 and CTL proliferation. Furthermore, co-culturing AuNP-pretreated BMDCs with OVA-specific CD4+ and CD8+ T cells increased T cell proliferation and augmented IFN-γ production from CD8+ T cells and IL-2 secretion by both T cell subsets. These findings indicate that AuNPs can serve as effective vaccine adjuvants by inducing Th1 and CTL responses [192]. In a study by Zhang et al. focused on the CD8 T cell response induced by AuNPs coated with multilayer polyelectrolytes, it was observed that these AuNPs increasingly activated DCs. This increased activation was marked by elevated levels of CD86, CD80, CD40, TLR3 signaling, antigen presentation, and, consequently, an enhanced antigen-specific T CD8 response [193].

#### 7.1.2. Effect of AuNP Size on T Cells

AuNPs can reportedly influence DCs’ induction of T cell differentiation into Th17 cells. Specifically, 50 nm AuNPs (but not 10 nm) were found to favor Th17 polarization. In addition, it was reported that 50 nm AuNPs promoted Th1 cell proliferation, whereas 10 nm gold NPs enhanced DCs’ capacity to promote Th2 polarization. Interestingly, 10 nm AuNPs exhibited an immunosuppressive effect by increasing IL-10 secretion and inhibiting LPS-induced IL-12p70, while the larger counterpart (50 nm AuNPs) stimulated a Th17 response. Moreover, the efficient internalization of both AuNPs resulted in reduced expressions of CD83 and CD86, suggesting DC activation without further maturation [88].

In a study by Fernandez et al., it was found that the internalization of 12 nm AuNPs by DCs led to a strong proliferative response in NK cells and CTLs. In contrast, 2 nm zwitterionic AuNPs induced an immunosuppressive response favoring Tregs [176]. Similarly, Guevel et al. demonstrated that 12 nm AuNPs induced DC maturation and significantly increased IL-12 and IFN-γ cytokine secretion, promoting Th1 cell-mediated immunity compared to 2 nm AuNPs [166]. In another study, Dey et al. revealed that AuNPs with a diameter of 97.01 ± 7.29 nm altered the metabolic activity in macrophages, increasing mitochondrial glycolysis and respiration rate. Only a minimal metabolic effect was exerted on DCs. The AuNPs also increased IL-6, MCP-1, ROS, and TNF-α production. The antigen presentation capacity of both macrophages and DCs was augmented, leading to enhanced antigen presentation to T cells and the subsequent generation of potent Th1, Th2, and Th17 responses [100].

### 7.2. B Lymphocytes

Hočevar et al. studied the interactions between nanospheres and nanorods of PEG- and PEG/PVA-AuNPs with B lymphocytes. Their results indicated that none of the AuNPs reduced B cell viability. However, polymer-coated AuNPs exhibited weaker interactions with B cells compared to uncoated AuNPs. Additionally, uncoated nanospheres and nanorods decreased IL-6 secretion by plasma cells, suggesting a functional impairment. Nevertheless, nanosphere AuNPs were unaffected by the activation markers of naive B cells (CD69, CD86, and MHC II) and increased the secretion of IL-1β and IL-6 pro-inflammatory cytokines [194].

In addition, previous studies have demonstrated that DC-targeted AuNPs can enhance the efficacy of epicutaneous and sublingual immunotherapies through the targeted delivery of allergens to DCs. These studies indicate that AuNPs can activate tolerogenic DCs, and the effect on maturation and activation is possibly size-dependent. These tolerogenic AuNP-targeted DCs have been shown to shift T cell responses towards Th1-Treg, resulting in the suppression of Th2–Th17 responses, with increased concentrations of IL-10, IFN-γ, and TGF-β and decreased levels of IL-4 and IL-17A cytokines. Additionally, the responses of allergen-IgE B cells shift towards secretory IgG B cells [71,169]. Studies highlighting the impact of AuNPs on adaptive immune cells are summarized in Table 2.

## 8. Unraveling the Mechanisms Underlying Anti-Inflammatory Effects of AuNPs

As previously mentioned, numerous studies have indicated the anti-inflammatory potential of AuNPs. In this section, we will delve into the possible mechanisms underlying these effects. AuNPs exert anti-inflammatory and antioxidant actions through various mechanisms—by te reduction in pro-inflammatory cytokines (such as IL-1β, TNF-α, and IL-6), decreasing markers of oxidative tissue damage [123,188,195,196], and suppressing ROS generation [102] and NO production [183], as well as by modulating the MAPK and NF-κB signaling pathways [183].

### 8.1. Modulation of the NF-κB Pathway

AuNPs inhibit NF-κB activation, thereby deactivating inflammatory mediators across various cell types through various mechanisms. Reports suggest that AuNPs inhibit the DNA binding affinity of NF-κB [197] or block the TPA-induced nuclear translocation of NF-κB [198]. Additionally, it has been proposed that AuNPs may inhibit the activity of thioredoxin reductase (TrxR), a master regulator of NF-κB, thereby suppressing TNF-α-induced NF-κB-dependent gene expression [199]. Another mechanism involves the significant inhibition of JAK1/STAT3 phosphorylation, subsequently blocking JAK1/STAT3 signaling [200].

**Figure 4:**
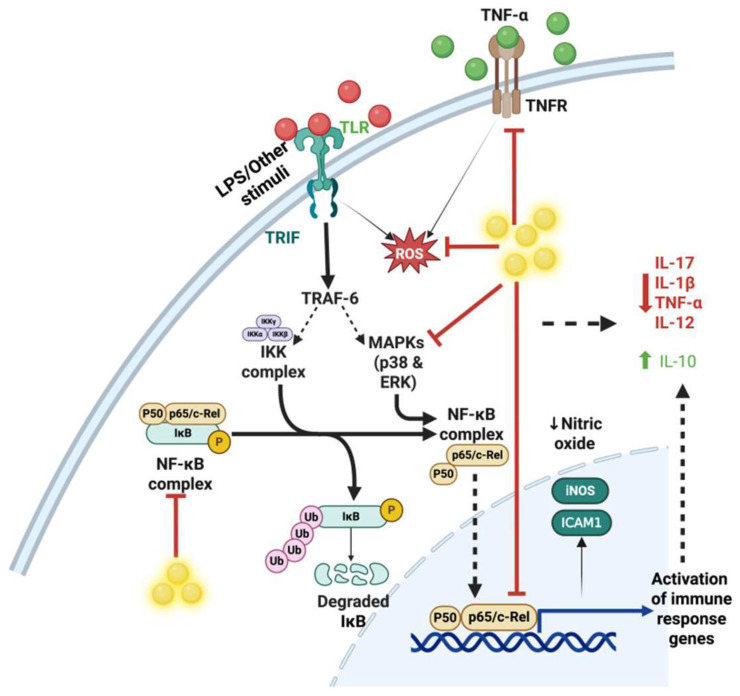
NF-κB pathway inhibition. Lipo-polysaccharide or other stimuli activating toll like receptors (TLRs) result in activation and translocation of NF-κB to the nucleus and upregulation of pro-inflammatory genes while downregulating anti-inflammatory genes. AuNPs inhibit NF-κB activity directly to the nucleus and also the TPA-induced translocation into the nucleus. By inhibiting the IκKβ, AuNPs further diminish NF-κB activation. Furhter, by sequestering HMGB-1 and other cytokines, AuNPs prevent TNFR and TLR signaling. They also modulate MAPK pathway to exert anti-inflammatory effects. AuNPs also scavenge ROS by adsorbing ROS on the surface.

Jeon et al. demonstrated that AuNPs could inhibit NF-κB activation by modifying cysteine-179 of the Ikappa B kinase subunit (IκKβ), blocking its activity, and reducing the production of pro-inflammatory mediators [201]. NF-κB regulates the transcription and production of numerous inflammatory cytokines, and its inactivation results in altered gene expression and the subsequently reduced production of inflammatory mediators across different cell types [202]. Ma et al. found that 10 and 15 nm AuNPs inhibited iNOS expression and subsequently NO production by blocking NF-κB and STAT1 activation in LPS-stimulated RAW 264.7 cells. NF-κB and STAT1 inhibition was mediated by the suppression of Akt (Protein kinase B) activity and also the downregulation of IFN-β [183]. Interestingly, in a study by Nishanth et al., NPs like silver (Ag) induced the significant production of pro-inflammatory mediators (COX-2, IL-6, and TNF-α) by stimulating the nuclear translocation of NF-κB subunits (p65/p50) in RAW 264.7 cells. Surprisingly, AuNPs failed to activate NF-κB or induce the translocation of NF-κB subunits to the nucleus. Furthermore, AuNPs failed to induce any IL-6 release or generate ROS [185] (Figure 4).

### 8.2. Modulation of MAPK and PI3K Pathways

The MAPK and PI3K pathways are other signaling pathways that regulate the transcription and translation of inflammatory cytokines. Inactivation of these pathways can lead to reduced pro-inflammatory gene expression and subsequently limit inflammation [202]. Some studies have shown that AuNPs modulate these signaling pathways and downregulate the production of proinflammatory cytokines and ROS. Carvalho et al. reported that small (~7.4 nm diameter) AuNPs exhibit anti-inflammatory and antioxidant activity by downregulating hepatic Kupffer cell and stellate cell activity. The study noted that AuNPs significantly reduced pro-inflammatory cytokines (IL-1β and TNF-α) and markers of oxidative stress (SOD-1 and GPx-1) in a rat model of liver injury. The anti-inflammatory effects were mediated through the modulation of the PI3K/protein kinase B (PI3K/Akt) and MAPK pathways [203].

More recently, Goa et al. reported that gold clusters could suppress the production of inflammatory mediators from macrophages stimulated by LPS. NF-κB activation, IκBα, and MAPK (p38, ERK, and JNK) pathway phosphorylation were suppressed by the clusters in a dose-dependent manner. These findings suggest that gold clusters exhibit anti-inflammatory effects by downregulating NF-κB and MAPK activation [204]. Another study demonstrated a significant reduction in pro-inflammatory cytokines (TNF-α and IL-1β), chemokines (CX3CL1, CXCl10, and CCL8), intercellular adhesion molecule (ICAM), and enzymes such as the matrix metalloproteinase-2/9 (MMP-2/9), COX-2, and ROS/NOS in cells treated with AuNPs in a dose-dependent fashion. The results indicated that AuNPs reduced NF-κB activation through ERK1/ERK2/MAPK/Akt/tuberin-mTOR pathways, targeting inflammatory genes and cellular stress responses [127]. These mechanisms indicate that AuNPs can inactivate transcription factors, leading to changes in protein expression.

### 8.3. IL-1β Trapping

**Figure 5:**
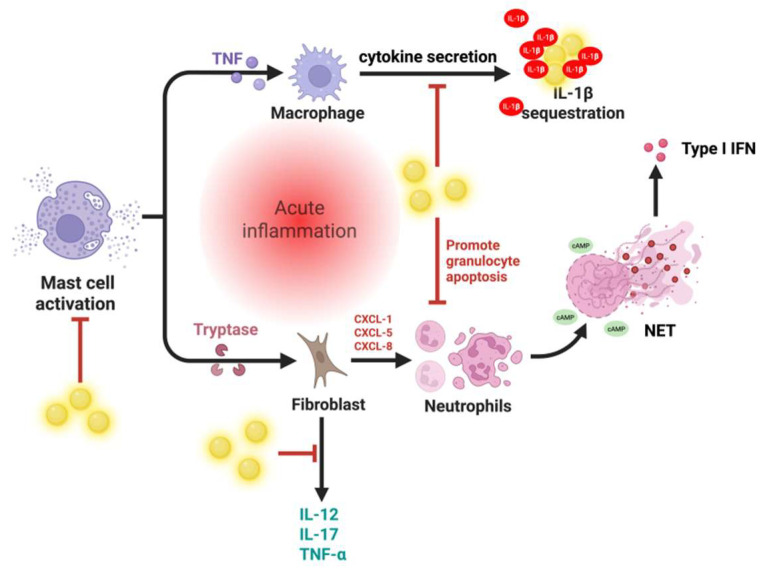
AuNPs limit acute inflammation. AuNPs dampen inflammation by inhibiting mast cell degranulation on prolonged incubation and possibly prevent IgE–FcεRI crosslinking preventing granule exocytosis. AuNPs can trigger NETosis, leading to greater trapping of AuNps, which go on to cleave cytoskeletal proteins and induce ER stress, promoting neutrophil apoptosis and inhibiting macrophage function. AuNPs, especially smaller sizes, owing to their large surface area, can sequester cytokines (IL-1β), preventing amplification of inflammatory process.

In vitro and in vivo studies by Sumbayev et al. revealed that citrate-stabilized AuNPs of varying sizes (5, 15, 20, and 35 nm), in a size-dependent manner, not only failed to activate the inflammasome pathway in THP-1 cells, but also downregulated the inflammatory process by selectively inhibiting the IL-1β-dependent pathway. The anti-inflammatory activity of AuNPs was attributed to the interference of inflammatory signal transmission via extracellular interactions with IL-1β. The aggregation of IL-1β around the AuNPs nidus reduced the likelihood of IL-1β-interleukin-1 receptor (IL-1R) binding, thereby inhibiting subsequent signaling pathways. Notably, 5 nm AuNPs exhibited the most potent inhibitory effect (approximately a 2-fold decrease in TNF-α secretion) among the different sizes. The high specific surface area of the ultra-small AuNPs possibly resulted in their greater affinity for IL-1β [123] (Figure 5).

### 8.4. Interference with TLR Signaling

Research has demonstrated that AuNPs of all sizes, including 4, 11, 19, 35, and 45 nm, exhibit the ability to suppress TNF-α production by macrophages when stimulated by CpG. This suppression is particularly pronounced in the case of 4 nm AuNPs, which possess a small size. AuNPs achieve this effect by exerting potent modulatory activity within lysosomes. Following phagocytosis, the AuNPs bind to the high mobility group box 1 (HMGB-1). This interaction inhibits the activation of TLR9, subsequently weakening downstream signaling and stopping TNF-α production. The heightened affinity of small AuNPs for HMGB-1, driven by their greater cellular uptake and higher specific surface area, likely contributes to their effectiveness in modulating TLR9 function. These signal modulations related to TLR9 and HMGB-1 functions need to be taken into account when assessing the safe application of AuNPs in biomedical contexts [188].

Zhu et al. evaluated citrate- and PVP-stabilized 5 nm AuNPs, as well as tannic acid (TA)-stabilized AuNPs, in sizes ranging from 5 to 60 nm in RAW 264.7 cells. The results showcased that 5 nm AuNPs with all three different coatings exhibited anti-inflammatory activity compared to the large-sized TA-coated AuNPs. They inhibited LPS-triggered iNOS and NO production by reducing TLR4 and also inhibited the expression of pro-inflammatory cytokines (IL-6 and TNF-α) by blocking NF-κB activation through both TLR4 reduction and the catalytic detoxification of hydrogen peroxide and peroxynitrite free radicals [187]. Moreover, another study by Gao et al. showed that peptide–AuNP hybrids exhibited size-dependent anti-inflammatory effects by potently inhibiting TLR4 signaling and the subsequent production of downstream pro-inflammatory cytokines (CCL2, and CCL4). This was achieved by modulating endosomal acidification both in vitro and in vivo. AuNPs with a 20 nm core size exhibited the highest cellular uptake and the strongest endosomal pH buffering capacity, which contributed to a significant reduction in TLR4 activation compared to smaller hybrid AuNPs with 5 nm and 3 nm core sizes. This study suggests that NP size influences the anti-inflammatory potency of peptide–AuNP hybrids. Thus, AuNPs could be a promising novel class of nanotherapeutics for modulating TLR signaling to treat conditions like acute lung injury (ALI) and acute respiratory distress syndrome (ARDS) [205]. Additionally, Chen et al. reported that 27 nm AuNPs could downregulate TLR4 and TNF-α in macrophages, improving lipid metabolic markers in the liver of mice fed a high-fat diet (HFD) [206,207].

### 8.5. Other Possible Mechanisms

Some studies have implicated the involvement of the cytokine IL-10 in the anti-inflammatory action of AuNPs. AuNPs reduce the proportion of CD14+/CD16+ monocytes and decrease the expression of CD86, HLA-DR, IL-12/IL-23, and TNF-*α* by these cells, primarily through an increase in IL-10 [186]. AuNPs have also been demonstrated to reduce ROS levels. ROS is an established activator of the NF-κB transcription pathway [208]. Therefore, ROS quenching may play an additional role in the effects of AuNPs on NF-κB activation. Despite the several studies that have demonstrated the anti-inflammatory effects of AuNPs, the exact mechanisms underlying these effects remain unclear. In conclusion, multiple pathways and mechanisms are involved in the effects of AuNPs, necessitating further research to explore the crosstalk of these pathways and investigate diverse mechanisms influencing the effects of AuNPs.

## 9. Therapeutic Applications of AuNPs in Inflammatory Diseases

The use of gold in traditional medicine dates back to ancient China and Egypt [209]. Au(I) compounds have been clinically approved therapies for RA since the 20th century, following the observations of effective treatment for rheumatoid arthritis made by Jacques Forestier [210]. However, their usage has recently declined due to the undesirable immune reactions experienced by some patients [211,212,213,214]. The susceptibility to many of these side effects is associated with variations in the MHC class of genes [214,215,216]. Despite a significant reduction in their use, salts like the gold sodium thiomalate continue to serve as valuable agents in the management of RA. Additionally, chrysotherapy, a treatment method using gold compounds, remains an important approach in RA therapy. Nevertheless, the prolonged administration of gold salts can have adverse or toxic effects [217]. In their unbound state, monovalent gold (Au^+^) drugs spontaneously disproportionate to generate trivalent (Au^3+^) and zero-valent (Au^0^) forms [218]. While the Au^0^ is the pharmacologically active component, Au^3+^ triggers significant side effects and is responsible for the toxicity observed during aurotherapy in RA patients [219,220]. Unsurprisingly, various studies have investigated AuNPs to mitigate these side effects.

The promising impact of AuNPs includes an excellent biocompatibility, non-toxicity, and non-immunogenic properties. AuNPs do not induce pro-inflammatory mediators, and their anti-inflammatory properties have encouraged further research. As mentioned earlier, their anti-inflammatory property is attributed to the downregulation of the transcription factor NF-κB and subsequent inflammatory mediators such as IL-1β, COX-2, and TNF-α. Table 3 lists the details of studies exploring a possible mechanism by which AuNPs exert systemic anti-inflammatory and pro-inflammatory effects.

**Table 3:** Mechanisms of systemic anti-inflammatory effects of AuNPs.

AuNPs Properties	Model/Cell Line	Mechanism of Actions	Main Effect		Ref.
			Inflammatory	Anti-Inflammatory	
27.3 ± 0.5 nm Low (0.0785 μg/g/day), Medium (0.785 μg/g/day) High (7.85 μg/g/day)	HFD mice model	- ↓TLR4 - ↓TNF-α expression (low and medium) - ↓F4/80 (medium)		✔	[206,207]
6.3 nm	Murine model of atopic asthma	- ↓IL-1, IL-5 and IL-6 levels in BAL - ↓IL-4, IL-5, IL-6, IL-13, eotaxin-1, and eotaxin-2 in lung tissue - ↓Airway inflammatory infiltrates - ↓MDA levels		✔	[221]
20 nm	Rat model of sporadic Alzheimer’s dementia	- ↓In IL-1β and NF-κβ levels - Prevent STZ induced neuroinflammation and oxidative damage by ↑SOD, and GPX activity		✔	[222]
20 and 45 μm	Mice model for brain injury	- ↓Cerebral TNF-α levels, - ↑Oxidative DNA damage and pro-apoptotic markers (cleaved caspase-3, cytochrome c leakage)		✔	[223]
25 nm	Mouse model of EAE	- ↑IL-27 secretion (dose-dependent) - ↓CNS leukocyte infiltration and demyelinated foci - ↓IL-23		✔	[224]
7.4 ± 2.8 nm	Swiss mice	- 49.3% ↓ in leukocyte migration - ↓IL-1β and TNF-α - ↓Peripheral analgesia and inflammation		✔	[195]
5, 20, and 50 nm	Mice	- 5 nm: delayed ↑in IL-1β and IL-6 mRNA expression (Day 7) in mouse brain - 20 and 50 nm: ~pro-inflammatory cytokines - ~TNF-α expression	✔ (small sizes)		[113]
20 nm	SAECs	- ↑Serum amyloid A (SAA) and TLR2, and ↑NF-κβ activation	✔		[225]
15 nm	A549	- ~mRNA expression of TNF-α, IL-8 and iNOS, or antioxidant (HO-1 and SOD2) markers - ~protein expression (IL-1β, IL-2, IL-4, IL-6, IL-8, IL-10, GM-CSF, INF-γ, and TNF-α)	~	~	[226]
50 and 250 nm	Rat model of pulmonary inflammation	- 250 nm: ↑IL-6 and TNF-α - Agglomerated 50 nm: ↑TNF-α	✔ Mild inflammatory reaction		[227]
16–25, 40 (400 μg/kg)	DNBS-colitis mice model	- ↓In the IL-6 and TNF-α levels - ↓CAT, GSH, and SOD levels in the colon		✔	[228]
13 nm	Rat CIA model	- ↓Leukocyte and macrophage infiltration - ↓IL-1β, VEGF, and TNF-α		✔	[229]
35 nm	Rat model of tendinous injury	- ↓IL-1β and TNF-α		✔	[117]
13 or 50 nm	CIA mice model	- 50 nm: ↓inflammatory infiltration (lymphocytes, leukocytes, macrophages) - 13 nm: ↓macrophage and lymphocyte infiltration		✔	[115]
10 nm	Rat Animal model of Tendinitis	- ↓IL-1β and TNF-α levels		✔	[230]
25 nm	Experimental rat model of muscle overuse	- ↓IL-6 and TNF-α - ↓SOD and GPX activity - ↓O_2_^−^ and NO_2_^−^ levels		✔	[231]
21.3 ± 0.7 nm (12.5, 25, and 50 ppm)	Sepsis mouse model	- ↓IL-1β, IL-6, and TNF-α (6 h and 24 h) - ↓IL-10 secretion at 6 and 24 h. - ↓M1 polarization (↑CD86^+ve^ ↓iNOS and Nur77) - ↑M2 polarization (↑CD206^+ve^ ↑Arg1 and PPARγ)		✔	[161]
10 nm and 50 nm	Wistar rats	- Liver: 50 nm AuNPs ↑IL-1β, IL-6, and TNF-α gene expression on D1(decrease by D6) - Kidney: ~IL-1β expression - Systemic: 50 nm ↑IL-6 and TNF- α expression on D1 (decrease by D5)	✔ (Transient inflammatory effects)		[157]
30 nm	Wistar rats	- Modulate TLR4-NF-κβ pathway - ↓Intraocular inflammation and oxidative damage		✔	[232]
20 nm	Wistar rats	- ↓IL-1β - ↓COX-2, iNOS, NF-κβ, and TNF-α mRNA expression		✔	[117]
25–50 nm	NHDF and NHEK	- ↓IL-6, IL-12, and TNF-α - ↓VEGF and bFGF		✔ Anti-angiogenic activity	[233]
10–50 nm	Jurkat and U937	- ↑TNF-α synthesis - ↓IL-6 and IL-12		✔	[234]
5.5 nm	HUVEC and VEC	- ↓NF-κβ pathway - ↓TNF-α and ROS production - ↑Degradation of CAM proteins→ reduce monocyte adhesion to		✔	[235]
Gold nanorods (50 × 15 nm) Stabilized with surface-coupled peptides	Human primary reticuloendothelial cells	- ↑CCL2, CCL3, and CCL4 (macrophages) PEG-OH capped particles ↑chemokine secretion (DCs) - ↑CXCL9 (Macrophages), and inhibit in DC - GLF modification ↑IL-1β, - RGD modification ↓IL-1β - ↑IL-6 (DCs only) - ↑TNF-α (macrophages) by peptide-bound AuNPs - ~TNF-α (DCs)	✔ (AuNP-GLF)	✔ (AuNP-RGD)	[148]

(↓—Decrease ↑—Increase ~—No effect).

## 10. Mechanisms of AuNP Cytotoxicity

Analyzing AuNP toxicity in in vitro and in vivo systems is complex, as evidenced by the apparent controversial and often contradictory results reported in various studies. Broadly stated, the toxic effects of AuNPs are influenced by multiple factors, including their inherent physicochemical properties (size, shape, surface charge, surface chemistry, and aggregation state) [236], as well as the environmental conditions (normal organs, blood, inflamed tissues, and tumors), making it difficult to provide a generalizable result. While AuNPs have been found to be non-toxic in human leukemia cell lines [237] and immune cells [102], divergent data has been reported equally prominently, with toxicity noted in Cos-1 cells, red blood cells, and bacterial cultures such as E. coli [134]. The inherent heterogeneity of biological systems, encompassing diverse cell types and complex environmental variables, makes extrapolating data generated from lab research into the real world challenging.

### 10.1. AuNP Toxicity Is Mediated by ROS

While a consensus on the toxic potential of AuNPs is lacking, a large body of studies implicate AuNP-triggered ROS as a key driver of cellular and tissue damage. A significant proportion of toxicity evaluations have been carried out in human cancer cell lines—HeLa cervical cancer cells [238], MDA-MB-231 breast cancer cells [239], and HepG2 liver carcinoma cells [240]. In HCT-116 colon cancer cells, AuNPs have been shown to promote a pro-oxidant state by stimulating total oxidative stress, reducing total antioxidant capacity, and impairing key antioxidant enzymes (superoxide dismutase, glutathione peroxidase, and catalase) in a dose-dependent manner, thereby triggering apoptosis. Of note, concentrations as low as 50 µg/mL triggered significant oxidative imbalance and reduced cell viability, supporting their potential as pro-oxidant cancer therapeutics. Future work should explore the molecular pathways underlying ROS induction and extend evaluations to other cancer types and in vivo models [241]. In this case, the particles were citrate stabilized and, therefore, anionic in charge, partly explaining their intrinsic toxicity and ensuring that they would not be used clinically, since they are promptly opsonized and cleared from circulation upon intravenous administration. Similarly, bare, surface non-modified AuNPs have also been shown to damage benign cells in a similar fashion. Replacing the citrate buffer coating of AuNPs with serum does not make these particles less toxic, since they still accumulate in the endo-lysosomal compartment of cells of human lung fibroblasts in vitro, driving cytotoxicity via enhanced ROS production, resulting in lipid peroxidation and the formation of malondialdehyde-protein adducts, and accelerating autophagosome formation. This is further accompanied by upregulation in cellular stress response genes (COX-2 and PNK), indicating DNA damage and the activation of cellular defense pathways [242]. Notably, AuNPs coated with pleural membrane fluid demonstrated no cytotoxicity to human hepatoma cell line C3, but elicited toxic effects and increased cytokine production in primary rat hepatocytes, indicating that both nanoparticle surface modification and cell type significantly influence AuNP toxicity [243].

Apart from bare AuNPs, surface-functionalized AuNPs can also exert cellular toxicity depending on the surface chemistry. Triphenylphosphine sulfonate-capped AuNPs were shown to induce potent size- and ligand-dependent cytotoxicity through oxidative stress and resultant mitochondrial dysfunction, leading to necrosis rather than apoptosis. The idea of ROS-mediated AuNP cytotoxicity is also supported by the fact that thiol-containing antioxidants (glutathione [GSH] and *N*-acetyl cysteine [NAC]) were able to mitigate cell damage. In contrast, ascorbic acid, a non-thiol-containing anti-oxidant, was unable to quench oxidative stress, emphasizing the critical role of surface chemistry in AuNP toxicity [244].

Different surface coatings also alleviate ROS-induced cytotoxicity. AuNPs with different surface coatings (PEG, low-molecular-weight (LMW) PEI, and high-molecular-weight (HMW) PEI) exhibited size- and coating-dependent cytotoxicity in HepG2 cells. While all formulations increased ROS, only PEI-HMW-coated AuNPs triggered significant oxidative stress, indicating that surface chemistry influences cytotoxic potential. PEGylation, conversely, had minimal toxicity [245]. Also, chitosan-coated 3–10 nm AuNPs exhibited selective size- and coating-dependent cytotoxicity in leukemic CEM and K562 cells, while sparing healthy PBMCs and BM cells. Cytotoxicity was ROS-mediated, involving mitochondrial membrane depolarization, DNA damage (as assessed by γH2AX foci), and the activation of distinct cell death pathways—caspase-dependent apoptosis in CEM and necroptosis in K562. These AuNPs also triggered protective autophagy, suggesting surface chemistry and redox imbalance as key modulators of AuNP-induced leukemic cell death [246].

### 10.2. Effect of AuNP Size on ROS Production

The extent of oxidative damage exerted by AuNPs is also influenced by particle size. Small gold nanorods (AuNRs), 10 and 25 nm in diameter, coated with PEG, demonstrated size- and dose-dependent cytotoxicity in human HepG2 liver cells following prolonged exposure for 48 h. The smaller 10 nm GNRs exhibited greater toxicity, evidenced by decreased cell viability, increased lactate dehydrogenase leakage, and the induction of oxidative stress markers and IL-8 release, culminating in caspase-3 activation [247]. Little data on characterization of the AuNRs was provided, assessing how much surfactant (presumably CTAB) remained on the surface of the rods after PEGylation and how much the PEG reversed the cationic surface charge of the bare AuNRs. Medium- to large-sized AuNPs (30, 50, and 90 nm) are also known to exert size- and dose-dependent cytotoxicity in human HL-60 leukemia cells and HepG2 cells, largely driven by oxidative stress—evidenced by GSH depletion, ROS elevation, and reduced SOD activity. Such effects are more pronounced in the leukemic cells. Even though the smaller particles triggered maximal oxidative stress (reduced antioxidant levels of GSH and SOD and elevated ROS), it could be partially mitigated by NAC. Conversely, NAC was unable to minimize oxidative damage from larger AuNPs [248].

### 10.3. Protein Corona Significantly Modulates AuNP Cytotoxicity

The cytotoxic effects of AuNPs are significantly modulated by protein corona formation. In systemic circulation, the AuNP surface is passively enveloped by a negatively charged layer of plasma proteins, held in place by electrostatic forces. In human hepatocytes, bare branched PEI-coated AuNPs induced strong time- and concentration-dependent cytotoxicity linked to elevated reactive oxygen and nitrogen species (ROS/RNS) and the inhibition of CYP1A2, CYP2C9, and CYP3A4 production. Conversely, protein corona-coated AuNPs—especially those with human plasma or albumin—had reduced ROS/RNS generation, cellular uptake, and overall cytotoxicity, emphasizing how protein corona formation critically modulates AuNP cytotoxicity by altering nanoparticle surface properties, cellular uptake, and oxidative stress response [249].

In a single-cell-based, high-dimensional mass cytometry approach investigating the impact of serum-derived protein corona on the cellular effects and cytotoxicity of branched PEI-coated AuNPs in human peripheral blood mononuclear cells (PBMCs), fetal bovine serum (FBS)-derived protein corona formation significantly reduced AuNP cytotoxicity and cellular uptake across diverse immune cells (monocytes, dendritic cells, B cells, NK cells, and T cells) by altering nanoparticle surface functionality and stabilizing particle size [250]. However, in serum-free conditions, AuNPs caused higher cytotoxicity, especially in phagocytes (monocytes and dendritic cells). Increasing FBS concentrations (up to 10%) reduced cytotoxicity across all immune cell subtypes, even though different subsets utilize varied endocytic pathways (e.g., phagocytosis, receptor-mediated endocytosis, and micropinocytosis) for NP internalization. The protein corona stabilized the AuNP size and prevented agglomeration in addition to neutralizing the positive surface changes, resulting in reduced phagocytic uptake and thereby directly reducing toxicity in a dose-dependent fashion. The toxic effects in non-phagocytic cells (B, T, and NK cells) in low- or no-serum experimental conditions were primarily associated with high NP loads, suggesting that both uptake mechanism and protein corona coverage critically modulate toxicity [250]. Furthermore, phenotypic differences within T cell subsets (e.g., CD4+ vs. CD8+, and within CD8+ naïve killer T cell subsets) led to distinct patterns of AuNP uptake and cytotoxicity.

Importantly, the surface properties of AuNPs modulate the kinds of proteins that envelop them in circulation. Citrate-stabilized AuNPs are readily opsonized in circulation by a protein corona that increases their hydrodynamic diameter significantly from about 30 to 50 nm, and comprise all components of the serum complement family that facilitate phagocytosis by circulating and resident macrophages and macrophage-like cells of the reticuloendothelial system [251]. PEGylation reduces the total bound protein quantity but only minimally affects the composition of the protein corona. In total, 20 kDa PEG seems to provide more shielding from protein corona formation than lower-molecular-weight PEGs [252].

Collectively, these results highlight the crucial role of protein corona formation in modulating nanoparticle–cell interactions and their subsequent biological responses and underscore the need to evaluate AuNP toxicology in physiological contexts across biological systems. A comprehensive understanding of AuNP-induced adverse effects requires a systematic investigation of how different cell types, particularly immune cells, respond to variations in nanoparticle size, morphology, surface properties, concentration, and exposure duration. Excessive negative or positive surface charge (with zeta potentials beyond −10 mV and +10 mV) likely result in greater opsonization and clearance from circulation, and PEGylation decreases the quantity of bound proteins within the protein corona, increasing circulation time and likely reducing toxicity.

## 11. Conclusions

As outlined in the foregoing sections, AuNPs exert anti-inflammatory effects by hindering the secretion of pro-inflammatory cytokines, reducing inflammatory downstream signaling within immune cells, and inhibiting the immune roles of neutrophils, monocytes/macrophages, dendritic cells, mast cells, and T cells to modulate innate and adaptive immune responses. Merely assessing the cytotoxic effects of AuNPs fails to provide a comprehensive understanding of their potential impact on humans, especially with long-term or chronic exposure. It should be noted that while the systemic administration of AuNPs results in significant anti-inflammatory activity, gold is not a biodegradable material and may persist in the body for an extended period. Therefore, it is crucial to conduct precise analyses of their functional aspects to accurately determine the biological changes arising from the presence of AuNPs.

A thorough comprehension of the effect of AuNPs on immune cells, particularly APCs such as neutrophils, DCs, and macrophages, offers valuable insights into the potential adverse consequences of AuNP exposure. Evaluating the immunotoxicity of AuNPs should certainly focus on APCs, since these cells are involved in both nonspecific innate defenses and specific adaptive immune responses.

The uptake of AuNPs by immune cells and their fate within cells is governed by the core material and also by various physicochemical parameters, including charge, size, surface chemistry, and the nature of molecules present on the particle surface. These parameters also influence the potential of AuNPs to trigger any immune response or remain unrecognized by the immune system. Therefore, controlling the surface properties of engineered AuNPs is crucial in managing their uptake and impact on different organ systems when used for biological or medical applications, as well as any scenarios that may expose organisms to AuNPs. Consequently, the size and dosage of AuNPs can influence the internalization, accumulation, and toxicity of these NPs, potentially affecting their fate in biological environments, with smaller AuNPs possibly having more pronounced anti-inflammatory effects.

Currently, our understanding of how AuNPs modulate intracellular molecular pathways in immune cells remains quite limited. Although AuNPs may appear to have negligible effects, they can induce discrete functional modifications among different immune cell subsets. The interactions between AuNPs and intracellular molecules are likely influenced by physicochemical properties, especially the surface chemistry of AuNPs. Investigations into the structure–activity relationship are essential for rationally designing AuNP-based therapies and harnessing their immunomodulatory potential.

## Figures and Tables

**Table 2:** The inflammatory and anti-inflammatory effects of AuNPs on adaptive immune cells.

Immune Cells	AuNPs Properties	Model/Cell Line	Mechanism of Action	Main Effect		Ref.
				Inflammatory	Anti-Inflammatory	
T cells	PDDAC-, CTAB-, and PEI-modified Au nanorods (60 × 15 nm)	BALB/c mice	- ↑DC maturation - ↑APC co-stimulator molecules - ↑T CD4+ and CD8+ proliferation - ↑Th2 responses	✔		[190]
	50 and 10 nm	DCs and T cells (PBMCs)	- ↑DC activation - 50 nm—↑Th1 and Th17 differentiation and cytokine secretion - 10 nm AuNP—↓IL-12p70, ↑Th2 differentiation and ↑IL-10	✔ (50 nm)	✔ (10 nm)	[88]
	Dextran-modified AuNPs (~210–305 nm)	Splenocytes (OT-I and OT-II mice); BMDCs	- ↑Antigen presentation (BMDCs) - ↑MHC-I, MHC-II - ↑CD40, CD80, CD86 - ↑CTL and Th1 responses	✔		[192]
	Polyelectrolyte-coated AuNPs (~100–200 nm)	Splenic CD11c + DCs (B6 mice); Splenocytes (OT-I and OT-II mice)	- ↑DCs activation and antigen presentation - ↑CD86, CD80, CD40, TLR3 signaling - ↑Antigen-specific T CD8 response	✔		[193]
	2 and 12 nm	PBMCs, human monocyte-derived DCs	- 12 nm: ↑NK cell proliferation, IL-12 and IFN-γ cytokines, Th1 and CTLs responses - 2 nm: ↑Uptake; mild immunosuppression	✔ (12 nm)	✔ (2 nm)	[176]
	2 and 12 nm	PBMCs; human monocyte-derived DCs	- 12 nm: Th1 cell-mediated immunity, inflammatory NK cells - 2 nm: ~DC maturation	✔ (12 nm)	✔ (2 nm)	[166]
	AuNP-Allergen (15 nm)	BALB/c mice	- ↑IFN-γ and TGF-β - ↑Th1-Treg polarization - ↓IL-4		✔	[169]
	AuNP-Allergen (15 nm)	BALB/c mice	- ↑IFN-γ, IL-10, and TGF-β - ↑Th1-Treg polarization - ↓Th2-Th17 polarization - ↓IL-4 and IL-17A secretion		✔	[71]
	97.01 ± 7.29 nm	BMDCs (C57BL/6 mice); CD4+ (OT-II mice); J774.1A	- ↑IL-6, MCP-1, ROS, and TNF-α and antigen presentation capacity (MQs and DCs) - ↑Mitochondrial respiration and glycolysis (MQs) - ↑Th1, Th2, and Th17 responses	✔		[100]
B cells	PDDAC-, CTAB-, and PEI-modified Au nanorods (60 × 15 nm)	BALB/c mice	- ↑IgG2a production	✔		[190]
	PEG- and PEG/PVA-AuNPs	B lymphocytes (human Buffy coats)	- Uncoated nanospheres and nanorods AuNPs ↓IL-6 secretion		✔	[194]
	AuNP-Allergen (15 nm)	BALB/c mice	- allergen-IgE B cell secretory IgG B cells		✔	[169]
	AuNP-Allergen (15 nm)	BALB/c mice	- Change allergen-IgE B cell responses to secretory IgG B cells		✔	[71]

(↓—Decrease ↑—Increase ~—No effect).

## Abbreviations

AGIP	Amyloid growth inhibitory peptide
AgNPs	Silver nanoparticles
ALI	Acute lung injury
ALT	Alanine transaminase
APCs	Antigen-presenting cells
APPs	Acute-phase proteins
APR	Acute phase response
ARDS	Acute respiratory distress syndrome
Arg1	Arginase 1
AST	Aspartate transaminase
AuNPs	Gold nanoparticles
BBB	Blood–brain barrier
BMDCs	Bone-marrow-derived DCs
CIA	Collagen-induced arthritis
COX-2	Cyclooxygenase-2
CpG	Cytosine–phosphate–guanosine oligodeoxynucleotides
CTAB	Cetyltrimethylammonium bromide
DCs	Dendritic cells
ER-stress	Endoplasmic reticulum stress
GM-CSF	Granulocyte macrophage-colony stimulating factor
HFD	High-fat diet
HIF-1α	Hypoxia-inducible factor-1alpha
HMGB-1	High mobility group box 1
IBD	Inflammatory bowel disease
ICAM	Intercellular adhesion molecule
IFNs	Interferons
IκKβ	Ikappa B kinase-beta
IL	Interleukin
ILCs	innate lymphoid cells
IL-R	Interleukin receptor
iNOS	Inducible nitric oxide synthase
JAK	Janus kinase
MAPK	Mitogen-activated protein kinase
MCP-1	Monocyte chemotactic protein-1
MDA	Malondialdehyde
MMP-2/9	Matrix metalloproteinase-2/9
MQs	Macrophages
MUC1	Mucin 1
NADPH	Nicotinamide adenine dinucleotide phosphate
NETs	Neutrophil extracellular traps
NF-κB	Nuclear factor-kappa B
NK	Natural killer
nm	Nanometers
NO	Nitric oxide
NOS	Nitric oxide synthase
NPs	Nanoparticles
NSAIDs	Non-steroidal anti-inflammatory drugs
OVA	Ovalbumin
PDDAC	Poly-diallyl dimethylammonium chloride
PEG	Polyethylene glycol
PEI	Polyethyleneimine
PMA	Phorbol myristate acetate
PRRs	Pattern recognition receptors
PVA	Polyvinyl alcohol
PVP	Polyvinylpyrrolidone
RA	Rheumatoid arthritis
ROS	Reactive oxygen species
SAP	Sweet arrow peptide
SLE	Systemic lupus erythematosus
SPR	Surface plasmon resonance
STAT	Signal transducer and activator of transcription
TA	Tannic acid
TAMs	Tumor-associated macrophages
TLR	Toll-like receptor
TNF-α	Tumor necrosis factor-alpha
TrxR	Thioredoxin reductase
UC	Ulcerative colitis
VLPs	Virus-like particles
